# Plasma metabolites with mechanistic and clinical links to the neurovascular disease cavernous angioma

**DOI:** 10.1038/s43856-023-00265-1

**Published:** 2023-03-03

**Authors:** Abhinav Srinath, Bingqing Xie, Ying Li, Je Yeong Sone, Sharbel Romanos, Chang Chen, Anukriti Sharma, Sean Polster, Pieter C. Dorrestein, Kelly C. Weldon, Dorothy DeBiasse, Thomas Moore, Rhonda Lightle, Janne Koskimäki, Dongdong Zhang, Agnieszka Stadnik, Kristina Piedad, Matthew Hagan, Abdallah Shkoukani, Julián Carrión-Penagos, Dehua Bi, Le Shen, Robert Shenkar, Yuan Ji, Ashley Sidebottom, Eric Pamer, Jack A. Gilbert, Mark L. Kahn, Mark D’Souza, Dinanath Sulakhe, Issam A. Awad, Romuald Girard

**Affiliations:** 1 Neurovascular Surgery Program, Department of Neurological Surgery, The University of Chicago, 5841S. Maryland Avenue, Chicago, IL 60637 USA; 2 Department of Medicine, University of Chicago, Chicago, IL 60637 USA; 3 Department of Neurosurgery, First Affiliated Hospital of Harbin Medical University, 150001 Harbin, Heilongjiang China; 4 Bioinformatics Core, Center for Research Informatics, The University of Chicago, Chicago, IL 60637 USA; 5 Department of Surgery, The University of Chicago, 5841 S. Maryland Avenue, Chicago, IL 60637 USA; 6 Department of Pediatrics, The University of California San Diego and Scripps Institution of Oceanography, 9500 Gilman Drive, La Jolla, CA 92093 USA; 7 Department of Pharmacology, The University of California San Diego, 9500 Gilman Drive, La Jolla, CA 92093 USA; 8 Department of Public Health Sciences, University of Chicago, Chicago, IL USA; 9 Host-Microbe Metabolomics Facility, Duchossois Family Institute, University of Chicago, Chicago, IL USA; 10 Department of Medicine and Cardiovascular Institute, University of Pennsylvania, 3400 Civic Center Boulevard, Philadelphia, PA 19104 USA

**Keywords:** Diagnostic markers, Stroke, Metabolomics

## Abstract

**Background:**

Cavernous angiomas (CAs) affect 0.5% of the population, predisposing to serious neurologic sequelae from brain bleeding. A leaky gut epithelium associated with a permissive gut microbiome, was identified in patients who develop CAs, favoring lipid polysaccharide producing bacterial species. Micro-ribonucleic acids along with plasma levels of proteins reflecting angiogenesis and inflammation were also previously correlated with CA and CA with symptomatic hemorrhage.

**Methods:**

The plasma metabolome of CA patients and CA patients with symptomatic hemorrhage was assessed using liquid-chromatography mass spectrometry. Differential metabolites were identified using partial least squares-discriminant analysis (*p* < 0.05, FDR corrected). Interactions between these metabolites and the previously established CA transcriptome, microbiome, and differential proteins were queried for mechanistic relevance. Differential metabolites in CA patients with symptomatic hemorrhage were then validated in an independent, propensity matched cohort. A machine learning-implemented, Bayesian approach was used to integrate proteins, micro-RNAs and metabolites to develop a diagnostic model for CA patients with symptomatic hemorrhage.

**Results:**

Here we identify plasma metabolites, including cholic acid and hypoxanthine distinguishing CA patients, while arachidonic and linoleic acids distinguish those with symptomatic hemorrhage. Plasma metabolites are linked to the permissive microbiome genes, and to previously implicated disease mechanisms. The metabolites distinguishing CA with symptomatic hemorrhage are validated in an independent propensity-matched cohort, and their integration, along with levels of circulating miRNAs, enhance the performance of plasma protein biomarkers (up to 85% sensitivity and 80% specificity).

**Conclusions:**

Plasma metabolites reflect CAs and their hemorrhagic activity. A model of their multiomic integration is applicable to other pathologies.

## Plain Language Summary

Cavernous angiomas (CAs) are clusters of abnormal blood vessels found in the brain or spinal cord. A blood test that could identify people with CAs that have recently bled would help determine who need surgery or closer medical monitoring. We looked at the blood of people with CAs to compare the levels of metabolites, a type of small molecule produced within the body, in those who had recently bled and those who had not. We found that some metabolites may contribute to CA and have an impact on CA symptoms. Monitoring the levels of these metabolites can determine whether there had been a recent bleed. In the future, drugs or other therapies could be developed that would block or change the levels of these molecules and possibly be used to treat CA disease.

## Abstract (web summary)

Srinath, Xie et al. analyze plasma metabolites present in patients with cerebral cavernous angiomas. Cholic acid and hypoxanthine are found in those with Cavernous Angioma disease whilst arachidonic and linoleic acids are found in Cavernous Angioma patients with symptomatic hemorrhage.

## Introduction

Cavernous angiomas (CAs), also known as cerebral cavernous malformations, affect more than one million Americans^1^. They are neurovascular lesions characterized by dysmorphic dilated capillaries, or caverns, lined by a leaky endothelium prone to hemorrhage^2^. The only current treatments of CAs are surgical resection of symptomatic lesions, or radio-surgical ablation of lesions, which are associated with high morbidity as well as costs and are limited in cases of multiple brain lesions^2^. Hence, there is a strong interest in developing non-surgical therapies based on mechanistic pathobiology^3–5^.

About 30–40% of the cavernous angioma (CA) patients are familial and harbor a germline dominant mutation in one of three documented genes (*CCM1/KRIT1*, *CCM2/malcavernin*, or *CCM3/PDCD10*)^5^. Familial-CA patients develop new multifocal lesions throughout the brain during their life^2^. Sporadic cases manifest a solitary lesion, often associated with a developmental venous anomaly^2^. Both familial and sporadic CAs harbor somatic mutations of *CCM* genes in the lesional endothelium resulting in an indistinguishable lesion pathology^6–9^. In addition, CA patients can experience an aggressive clinical course with earlier clinical symptom onset, higher lesion burden, and more frequent symptomatic hemorrhage^10^.

The clinical course of CA disease remains highly variable^2,11^. Some patients live unfettered lives despite CA, while others could be severely disabled by the disease^2,11^. While the risk of an initial cavernous angioma manifesting symptomatic hemorrhage (CASH) remains low, approximately 0.08% per year, a CASH is more than tenfold more likely to rebleed with cumulative disability as compared to lesions that never bled^2,11,12^. Therefore, there is a need for accurate biomarkers to distinguish high risk cases, selecting them for invasive or novel interventions, and to track disease status, progression, and response to therapies^5,13^.

Several mechanisms have been shown to contribute to CA lesion genesis^5,14^. Multiple dysregulated pathways have been validated within the transcriptome of CA, including MAPK/MEKK3/ERK3, PI3K-Akt, and Notch. These dysregulated pathways affect blood–brain barrier permeability and endothelial tight junction stability^15^. An upregulation of the MAPK/MEKK3/ERK3 pathway, and the related KLF2 and KLF4 transcription factors, have been reported to increase RhoA kinase activity in preclinical murine models of CA disease, leading to destabilization of endothelial barrier function^14^. Of interest, an increase in lipopolysaccharide (LPS)-producing Gram-negative bacteria also stimulates MAPK/MEKK3/ERK3 activity via toll-like receptor 4 (TLR4) in brain microvascular endothelium, suggesting a role of the gut-brain axis in this disease^16^. Although many commensals are gram negative, an alteration in their levels might produce diseases. A leaky gut epithelium linked with a permissive gut microbiome has been associated with CA lesion genesis, and microbiome differences were shown in CASH cases^17^. In addition, disease severity has been linked to pro-inflammatory genotypes^18^, a lesional anticoagulant domain^19^, and more recently to somatic mutations of oncogenes in the same lesions^20–22^.

Several plasma proteins linked to the above mechanisms are being probed as potential biomarkers of CA^23^. While weighted combinations of plasma protein levels have shown promise as a biomarker of higher risk CASH cases^13,24,25^, diagnostic and prognostic associations have remained imperfect. Several plasma micro-ribonucleic acids (miRNAs) are differentially expressed (DE) in CASH patients, suggesting their possible use as biomarkers of hemorrhagic activity^13,25^, but their levels in the plasma have not been specifically investigated. The bacterial content of the gut microbiome also reflects various aspects of CA disease and may enhance biomarker associations in combination with plasma protein levels^17^. The analysis of an individual patient’s microbiome remains impractical for clinical assessment; there is therefore a need to identify other circulating molecules that may reflect the permissive CA microbiome, or other mechanistic aspects of CA pathogenesis.

Differential levels of circulating small-molecules (i.e., <1500 kDa) have recently been proposed as candidate markers of neurologic diseases, cancers and aging^26–28^. The profile of these molecules is referred to as the metabolome, and is thought to include substrates, intermediates, products, and waste derivatives of various physiological and metabolic cellular processes. These plasma metabolites have neither been investigated in CAs, nor have they been considered as candidate biomarkers in other cerebrovascular diseases.

Herein, the differential plasma metabolome was assessed in CA patients to investigate possible mechanistic links with the lesional transcriptome, as well as the differential gut microbiome and plasma proteome previously implicated in CA^29^. We hypothesize that (1) the plasma metabolome identifies molecules associated with the diagnosis of CA as well as its cardinal clinical manifestation of symptomatic hemorrhage (SH); (2) metabolites associated with clinical features are mechanistically related to genes implicated in CA disease and the permissive microbiome; and (3) we propose that multiomic integration of these metabolites with plasma protein and miRNA levels using Bayesian modeling, implemented using machine learning (ML) algorithms, may enhance previously proposed diagnostic associations with symptomatic hemorrhage, the critical clinical feature of this disease.

The analysis of the metabolome between CA patients and non-CA patients identifies 15 metabolites (*p* < 0.05, false discovery rate [FDR] corrected), including cholic acid and hypoxanthine that show interactions with the CA transcriptome, microbiome, and differential circulating proteins. In addition, the plasma levels of 4 metabolites (*p* < 0.05, FDR corrected), including arachidonic acid and linoleic acid were different between CASH and non-CASH patients. These results were further validated in an independent, propensity-matched cohort. In addition, an integrative diagnostic model of metabolites with circulating proteins and miRNAs was able to distinguish CASH patients from non-CASH patients with up to 85% sensitivity and 80% specificity. The development of sensitive and specific biomarkers can stratify patients for surgery and close medical intervention and could aid in the discovery of therapeutics.

## Methods

### CA patients and clinical parameters

For this prospective study, a discovery cohort of 53 consecutive CA patients (25 familial-CA, and 28 sporadic-CA) was enrolled at a single referral center (www.uchicagomedicine.org/ccm) between April 2017 and August 2018, during routine consultations or follow-up visits (Supplementary Table 1). The diagnosis of CA disease was established using clinical 3-Tesla MRI scanners by a senior neurosurgeon (IAA) with more than 30 years of experience in CA disease management. Patients with partial or complete resection of CA or any prior brain irradiation were excluded^10,23,24^.

In the discovery cohort, five patients experienced a CASH within the year preceding collection of the blood sample. A CASH is a cardinal event in the clinical course of CA disease^14^, and was defined as an overt hemorrhagic event, noted on diagnostic imaging, in conjunction with attributable neurological symptoms^30^. An independent cohort was later recruited to validate the metabolomic findings. One hundred and nine consecutive CA subjects, enrolled between August 2016 and October 2020 included 20 CASH cases. These were best matched with 20 of the non-CASH cases for (1) age at enrollment, (2) gender (male/female), (3) phenotype (sporadic/familial), and (4) harboring brainstem lesion (yes/no). A one-to-one mapping with the single nearest neighbor was performed to match CASH and non-CASH pairs using STATA version 16.0 (College Station, TX, USA). The 20 CASH and 20 non-CASH patients who were most closely matched were included as the validation cohort.

Finally, 17 healthy non-CA subjects were also concurrently enrolled. Subjects were included if they did not have (a) any medical or neurologic condition requiring ongoing follow-up or medical treatment in the preceding year, (b) a history of concussion or brain trauma in the preceding year, (c) a history of prior brain irradiation at any time, (d) been pregnant or lactating in the preceding year, (e) used recreational, psychoactive, or neuroleptic drugs in the prior year.

All subjects gave written informed consent in compliance with the Declaration of Helsinki, and the study was approved by the University of Chicago Institutional Review Board, which is guided by ethical principles consistent with the Belmont Report, and comply with the rules and regulations of the US Department of Health and Human Services Federal Policy for the Protection of Human Subjects (56 FR 28003).

### Whole blood collection and processing

Standard clinical 10 mL heparinized vacutainer tubes (BD Vacutainer, Becton, Dickinson and Company, Franklin Lakes, New Jersey, USA) were used to draw blood samples in conjunction with clinic visits. The plasma was isolated by centrifugation at 2300 rpm (1019 g) at 4 °C for 10 min (AllegraX-30R, Beckman Coulter, Brea, California, USA). Subsequently, 300 μL of supernatant plasma was aliquoted into 1.7 mL microcentrifuge tubes for storage at −80 °C. These aliquots were used to assess the plasma metabolome, as well as predefined proteins and miRNAs.

### Liquid-chromatography-tandem mass spectrometry data acquisition and analyses for plasma metabolites in the discovery cohort

For each sample, 30μL of plasma was thawed and mixed with 120μL of 100% methanol spiked with 1.25 μM sulfamethazine. The solution was homogenized for 5 min at 25 Hz using a Tissuelyser II (Qiagen, Hilden, Germany), and then stored at −20 °C for 2 h. Afterwards, samples were centrifuged at 14000 rpm for 15 min, and 120 μL of the supernatant was extracted and loaded in duplicate wells of a 96-wells plate. The plate was then concentrated using a CentriVap Benchtop Vacuum Concentrator (Labconco, Kansas City, USA) until dry, and stored at −80 °C until further analysis.

As part of liquid-chromatography-tandem mass spectrometry data acquisition (LC-MS/MS), samples were resuspended in 120μL of a 1:1 water: methanol mixture. Samples were then spiked with 1 μM of sulfadimethoxine, and transferred to a 96-wells autosampler plate. Plasma metabolome analysis was performed with an ultra-high-performance Thermo Dionex Ultimate 3000 UHPLC (ThermoFisher, Waltham, MA, USA) coupled to an ultra-high resolution quadrapole time of flight Bruker Daltonics MaXis HD (Billerica, MA, USA) mass spectrometer. Chromatographic separation was performed using a Phenomenex Kinetex column (Torrance, CA, USA). Two solvents were used: solvent A consisted of LC-MS grade water with 0.1% formic acid while the mobile phase (i.e., solvent B) consisted of LC-MS grade acetonitrile with 0.1% formic acid. For each sample, 5 μL of solution was injected into a flow rate of 0.5 mL/min using the following parameters for the gradient (1) 0–1 min at 5% of solvent B, (2) 1–11 min linear increase to 100% B, (3) 11–11.5 min at 100% B, and (4) 11.5–12 min linear gradient to 5% of solvent B. The data acquisition was performed using electrospray ionization in positive mode.

Data analyses were performed using a complete a lock mass correction using hexakis (1H, 1H, 2H-difluoroethoxy) phosphazene (Synquest Laboratories, Alachua, FL, USA) implemented within the Bruker Data Analysis Software. Feature detection was done using MZmine v2.38 software (http://mzmine.github.io/changelog.html)^31^. The parameters used were Centroid, Noise Level MS1 − 5.0E2 and MS2 − 5.0E1 (Mass Detection); MS Level 1, Minimum Time Span –0.02, Minimum Height – 1.5E3, m/z tolerance –0.02 m/z or 20 ppm (Chromatogram Builder); Algorithm – Local Min Search, Chromatographic Threshold – 0.01%, Search minimum in RT range –0.3 min, Minimum relative height –0.01%, Minimum absolute height –1.5E3, Min ratio of peak top/edge –2, Peak duration range –0.02–0.50 min, m/z center calculation – Median, m/z range for MS2 scan pairing (Da) –0.01, RT range for MS2 scan pairing (min) –0.1; Isotopic Peak Grouper: m/z tolerance –0.02 *m/z* or 20 ppm, Retention time tolerance –0.3 min, Maximum charge –4, Representative isotope – Most Intense (Chromatogram Deconvolution);m/z tolerance –0.02 m/z or 20 ppm, Weight for m/z –75, Retention time tolerance –0.3 min, Weight for RT –25; Gapfilling (peak finder): Intensity tolerance –20 %, m/z tolerance –0.005 m/z or 10 ppm, Retention time tolerance –0.2; Peak Filter: area –1.0E4 to 1.0E20 (Join Aligner). The MS2 file and quantification table were used in the Global Natural Products Social Molecular Networking feature based molecular networking workflow^32,33^ to get both networks and annotations for the metabolites. Discovery cohort metabolomic quantifications were performed at the Center for Microbiome Innovation at the University of California San Diego.

The MS2 file and quantification table were used in the Global Natural Products Social Molecular Networking feature based molecular networking workflow^32,33^ to get both networks and annotations for the metabolites. Discovery cohort metabolomic quantifications were performed at the Center for Microbiome Innovation at the University of California San Diego.

### Differential analyses of the plasma metabolome

The unsupervised differential metabolome was assessed with PLS-Discriminant Analysis (PLS-DA) using the R Software^34^. PLS-DA was utilized to determine linear combinations of candidate molecules for biomarker development, rather than a Student’s *t* test, which would show distinguishing power of an individual molecule. A random permutations analysis on the peak values of the differentially expressed metabolite was performed to estimate the significance of the computed PLS-DA coefficients. Only the peaks with significant PLS-DA coefficients (*p* < 0.05, FDR corrected) were selected. Normalized plasma concentrations of each metabolite were calculated for each patient. Unannotated peaks and peaks representing drug-related compounds were discarded. The supervised analyses to validate the metabolites of interest between CASH and non-CASH patients in the independent propensity matched cohort followed the same approach.

### Independent propensity-matched validation cohort

Fragmentation patterns of the differential plasma metabolites between patients with a CASH event in the prior year and non-CASH patients were validated in an independent propensity matched validation cohort using a supervised LC-MS/MS approach.

Metabolites were extracted using a 1:8 dilution (i.e., one volume of plasma for eight volumes of extraction solvent composed of 100% methanol spiked with internal standards). Plasma samples were extracted at −80 °C for 1 h. Samples were centrifuged at 20,000*g* for 15 min at −10 °C. Two-hundred µL of the supernatant was dried down under nitrogen stream at 1 L/min (bottom)−30 L/min (top) at 30 °C using the SPE Dry 96 Dual (Biotage, Uppsala, Sweden). The samples were then resuspended in 200 µL of 1:1 water:methanol diluent, and mixed at 1000 rpm for 15 min at 4 °C using a thermomixer (Eppendorf). Samples were then centrifuged at 20,000*g* for 15 min at 4 °C to remove insoluble debris, and 150 µL of extracted supernatant was taken through subsequent analysis on an Agilent 1290 infinity II liquid-chromatography system coupled to an Agilent 6546 QTOF mass spectrometer, equipped with an Agilent Jet Stream Electrospray Ionization source. The detection window was set to 100–1700 *m/z* with continuous infusion of a reference mass (Agilent ESI TOF Biopolymer Analysis Reference Mix) for mass calibration.

Bile acids were analyzed in negative mode and 5 µL of extracted supernatant was injected onto an XBridge BEH C18 Column (Waters Corporation, Milford, MA, USA) fitted with an XBridge BEH C18 guard (Waters Corporation) at 45 °C. The mobile phase A was water with 0.1% formic acid, while the mobile phase B was acetone with 0.1% formic acid. Gradient elution started with 28% of the mobile phase B with a flow rate of 0.4 mL/min for 1 min, and linearly increased to 33% over 5 min, then to 65% over 14 min. The flow rate was then increased to 0.6 mL/min, while the mobile phase B was increased to 98% over 0.5 min. These conditions were held constant for 3.5 min. Then, re-equilibration was performed for 3 min at a flow rate of 0.4 mL/min with 28% of the mobile phase B. The electrospray ionization conditions were set with the capillary voltage at 3.5 kilovolts (kVs), nozzle voltage at 2 kVsA ten-point calibration curve of glycodeoxycholic acid was generated, starting at a concentration of 100 µg/mL diluted in 1:1 water:methanol followed by nine 1:3 serial dilutions.

#### Fatty acids and others metabolites

The fatty acids and other metabolites of interest were assessed using 5 µL of the extracted supernatant injected onto an Acquity UPLC HSS T3 Column (Waters Corporation) fitted with an Acquity UPLC HSS T3 guard (Waters Corporation) at 45 °C. The mobile phase A was water with 0.1% formic acid and mobile phase B was 98% acetonitrile with 0.1% formic acid. Gradient elution started with 5% of the mobile phase B with a flow rate of 0.5 mL/min for 1 min, and linearly increased to 75% over 1 min, then to 100% over 8 min. Column washing was performed at 100% of the mobile phase B for 4 min. Re-equilibration was performed for 3 min with 5% of the mobile phase B. Fatty acids and bile acids were analyzed in negative mode with capillary voltage at 3.5 kVs and nozzle voltage at 2 kVs while other metabolites were analyzed in positive mode with nozzle voltage at 500 Vs. An additional Level 1 confirmation was completed to support the identification of linoleic acid using known standards of linoleic and rumenic acids (Supplementary Fig. 1).

All metabolites were separated and analyzed by ultra-high-pressure liquid-chromatography quadrupole time-of-flight (UHPLC-QTOF) using the 1290 infinity II liquid-chromatography system (Agilent, Santa Clara, CA, USA) coupled to the 6546 QTOF mass spectrometer (Agilent). The detection window was set at 100–1700 m/z with continuous infusion of the ESI TOF Biopolymer Analysis Reference Mix (Agilent) used for mass calibration for the assessments of bile and fatty acids as well as the other metabolites of interest.

All putative compound annotations from the discovery cohort were validated against authentic standards (MilliporeSigma, Darmstadt, Germany; Avanti Lipids, Alabaster, USA; Steraloids, Newport, USA) in the independent propensity-matched validation cohort. Authentic standards and samples were run on the same instrument and their retention times, detected *m/z*, and fragmentation pattern were validated for all compounds (Supplementary Data 1).

The data analyses were performed using MassHunter Profinder Analysis version B.10 software (Agilent) and confirmed by comparison with authentic standards. The metabolomic quantifications were performed at Host-Microbe Metabolomics Facility at the University of Chicago.

### Plasma protein assays

Plasma protein concentrations were determined using individual (ng/ml) enzyme-linked immunosorbent assay (ELISA) assays or multiplex (pg/ml) electrochemiluminescence immunoassays in accordance with manufacturer protocols^23–25^.

Individual ELISA assays were performed using commercially available immunoassay kits to assess interleukin-10 (IL-10), CRP, and sCD14 (R&D Systems, Minneapolis, Minnesota, USA).

All plates were washed with a BioTek 405TS plate washer (BioTek Instruments, Winooski, VT, USA), and absorbances were measured using a Bio-Rad iMark plate reader (Bio-Rad, Hercules, CA, USA). The assays were performed in the Neurovascular Research team laboratory at the University of Chicago.

Multiplex electrochemiluminescence immunoassays plates (Meso Scale Diagnostics, Rockville, MD, USA) were used to quantify VEGF. Multiplex electrochemiluminescence immunoassays were processed using a V-plex multi-spot assay (Meso Scale Diagnostics). Plate was washed with a BioTek 405TS plate washer (BioTek Instruments, Winooski, VT, USA), and plasma levels measurements were assessed using Bio-Rad MESO QuickPlex SQ 120 (Meso Scale Diagnostics), by measuring light signal when the electrochemiluminescent labels are stimulated by electricity^17^. The assessment was performed at the Flow Cytometry Core Facility at the University of Chicago.

In each plate, the plasma samples were loaded in parallel duplicate wells, and then averaged, as in ongoing protein biomarker development projects in our laboratory^13^. Plasma values greater ±2 standard deviations from the mean for each group were excluded as statistical outliers.

### Gut microbiome analyses

Gut microbiome analyses on a larger cohort of CA patients and healthy non-CA subjects was previously published by our group^17^. That report included gut microbiome data on the same patients enrolled herein in metabolome and plasma protein assays. The report also includes detailed methods of stool collection, 16S rRNA gene sequencing, metagenomics shotgun sequencing and analyses of bacterial abundance.

The 16S-sequencing libraries (Project ID = PRJEB35505) are available at the European Molecular Biology Laboratory-European Nucleotide Archive EMBL-ENA (https://www.ebi.ac.uk/ena/data/view/PRJEB35505).

The metagenomic shotgun sequencing libraries (BioProject ID = PRJNA629755) are available at the Sequence Read Archive at National Center for Biotechnology Information (https://www.ncbi.nlm.nih.gov/bioproject/PRJNA629755).

### Differential microbiome analyses and Kyoto Encyclopedia of Genes and Genomes (KEGG) pathways

Following 16S analyses of the differentially detected bacterial species, the corresponding genomes, ortholog genes, and pathways in CA and the disease sub-categories were extracted from the KEGG database using the REST API (https://www.kegg.jp/kegg/rest/keggapi.html). The python library KEGG-parser (version 0.0.1) was used to parse the queried data into a dictionary format. The annotation of metabolite origins via networks AMON library implemented in python (Python Software Foundation, Fredericksburg, VA, USA) was used to perform batch query and find reactions that involved genes differentially detected in the 16S experiment and metabolites detected in the metabolome experiment^35^.

Microbiome shotgun sequencing resulted as an abundance matrix between the UniRef90 (https://www.uniprot.org/) proteins within each detected microbiome species and CA patient samples. The Uniprot protein IDs were mapped through the KEGG REST API into the KEGG ortholog genes. The AMON python library was used for the batch query and verifying the reactions involving detected genes in the shotgun sequencing experiment and metabolites detected in the metabolome experiment^35^.

### Transcriptome and enriched-KEGG pathways analyses

The transcriptome of human lesional CA neurovascular units (NVUs) of surgically resected CA from 5 patients and autopsy samples from 3 healthy non-CA subjects has previously been published by our team^36^. NVUs were dissected using laser capture microdissection. Following, RNA isolation and sequencing were completed to determine genes differentially expressed in CA lesions compared to the NVU of healthy autopsy tissue. RNA was then extracted using an RNA isolation kit (RNeasy Micro Kit, Qiagen). cDNA libraries were generated using commercial low-input strand-specific RNA-Seq kits (Clontech) and sequenced on the Illumina HiSeq 4000 platform using single-end 50-bp reads (Illumina). The raw sequencing data (accession number = GSE123968) are available in the National Center for Biotechnology Information’s Gene Expression Omnibus database.

Differentially expressed gene (DEG) analyses were conducted using DESeq2, with an additive model for batch effect correction when necessary. DEGs were then classified into (1) upregulated, and (2) downregulated genes in CA patients (|Fold Change | >1.5; *p* < 0.05, FDR corrected). Using these two gene lists, enrichment analysis was generated with the web-based bioinformatic tool Lynx (http://lynx.ci.uchicago.edu)^37^ to extract two sets of over-represented KEGG pathways (*p* < 0.05, FDR corrected).

### Correlation between plasma protein levels and differentially expressed metabolites

The Pearson’s correlation was assessed based on the normalized metabolite measurement x and plasma protein concentration y from matched patients with both metabolome and plasma proteome data. The test statistic was based on Pearson’s coefficient cor(x, y) and followed a *t* distribution with length(x)−2 degrees of freedom, assuming the samples followed independent normal distributions. A two-sided test was used to estimate the p-value. The p-values were corrected for multiple tests from all the metabolite-plasma protein pairs using the FDR B&H method. All the above steps were completed using the R statistics library^34^.

### Integrative analyses of differential metabolome, proteome, and transcriptome of human CA disease

**Fig. 1:**
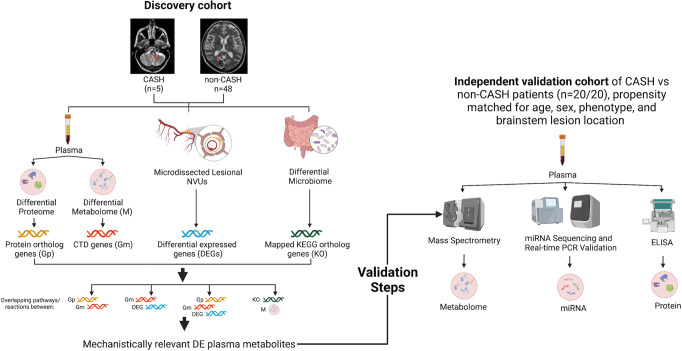
Methodology for analytic multi-omics integration of differential plasma metabolome and proteome, microbiome and lesional transcriptome, in cavernous angioma (CA) disease. A pipeline was implemented to study the integration of multiomic datasets of CA disease. The interacting Comparative Toxicogenomics Database (CTD) genes and their associated Kyoto Encyclopedia of Genes and Genomes (KEGG) pathways of the differential plasma metabolite (*p* < 0.05, FDR corrected; Bayes factor>3) were first identified and then analytically compared to the enriched-KEGG pathways of the differential plasma proteome, and lesional transcriptome (*p* < 0.05, FDR corrected; Bayes factor>3). The differential metabolites were then validated in an independent cohort, propensity matched for age, sex, brainstem lesion, and genotype. NVUs neurovascular units.

The interacting genes associated with the differential metabolites identified in the metabolome of CA patients (*p* < 0.05, FDR corrected) were queried using the Comparative Toxicogenomics Database (http://ctdbase.org/). The enriched-KEGG pathways containing at least one of the interacting genes associated with the differential metabolite were then identified using KEGG mapper (*p* < 0.05, FDR corrected; Bayes>3) (Fig. 1).

The enriched-KEGG pathways (*p* < 0.05, FDR corrected; Bayes Factor>3) associated with the upregulated and downregulated DEGs (*p* < 0.05, FDR corrected) within the human lesional transcriptome of NVUs (Gene Expression Omnibus #GSE123968)^36^ microdissected from CA lesions were queried independently using Lynx (http://lynx.ci.uchicago.edu)^37^. The common enriched-KEGG pathways between the differential metabolome and the lesional transcriptome were then identified (*p* < 0.05, FDR corrected; Bayes > 3) using KEGG mapper (https://www.genome.jp/kegg/tool/map_pathway1.html) (Fig. 1).

For the proteome (Supplementary Data 2), the coding genes of the plasma proteins with a documented role in CA disease were first queried using Uniprot (https://www.uniprot.org/) and Genecards (https://www.genecards.org/). KEGG pathway enrichment analysis was then performed using Lynx. Finally, the common enriched-KEGG pathways between the differential plasma proteome and metabolome were identified using KEGG mapper (*p* < 0.05, FDR corrected; Bayes>3).

The common enriched-KEGG pathways between the differential plasma metabolome and proteome as well as transcriptome were finally integrated to create a cross-referenced master pathway list.

### Integration of differential metabolome and microbiome of human CA disease

The genomes of the bacterial gut species showing different relative abundances in CA disease were queried using KEGG mapper (*p* < 0.05, FDR corrected; Bayes > 3)^17^. The genes identified were further mapped into KEGG ortholog genes using AMON software^35^ to extract reactions involving the ortholog genes and metabolites of interest. Shotgun sequencing was also queried to further support evidence for the presence of a reaction link (AMON) (Fig. 1).

### Plasma miRNome sequencing and differential expression analyses

Total RNAs from 100 μl of plasma were extracted using the miRNeasy Serum/Plasma Kit (Qiagen, Hilden, Germany) following the manufacturer’s recommendation^25,38^. cDNA libraries were then generated with commercially available Illumina small RNA-Seq kits (Clontech, Mountain View, CA, USA) and sequenced with the Illumina HiSeq 4000 platform (Illumina, San Diego, CA, USA) using single-end 50-bp reads, at the University of Chicago Genomics Core.

The differential expression analyses were conducted using R bioconductor package DESeq2, and considered significant at *p* < 0.05, FDR corrected. The in silico putative gene targets analysis of the differentially expressed (DE) micro-RNAs (miRNAs) was performed using miRWalk 3.0^39,40^. Gene targets were identified for the 3 different gene locations (3′ untranslated region [UTR], 5′ UTR, and coding sequence) using a random forest tree algorithm with a bonding prediction probability higher than 95%. Only putative gene targets that appeared in at least 2 of the 3 databases were considered^25,36,38^, and they were then queried within the transcriptome of human lesional CA NVUs^25,36^.

The DE plasma miRNAs were selected to be assessed using reverse transcription quantitative PCR (RT-qPCR) if they (1) were previously identified in preliminary analyses^25^, and/or (2) had putative gene targets within the transcriptome of human lesional CA related to at least 1 dysregulated KEGG pathway related to CA disease.

### RT-qPCR proof of feasibility

Relative and absolute quantifications of a panel of 6 pre-selected DE miRNAs were assessed using RT-qPCR. An exogenous spike-in control, *miR-cel-39-3p*, was added to all plasma samples prior to extraction and then measured to correct for extraction efficiency. The absolute quantification (i.e., number of miRNAs strands/μl) of each miRNA was estimated using a standard curve comprised of serial dilutions of known concentrations of the miRNAs of interest.

For the relative quantification, *miR-423-5p* was used as an endogenous control as it is expected to be expressed at equal levels across tissue types and samples^41^. This was validated using the NormFinder software, as *miR-423-5p* was shown to have the most stable expression across the controls tested (https://moma.dk/normfinder-software)^42^. For each miRNA, a relative quantification value greater than ±3 standard deviations away from the mean was defined as an outlier^43,44^. Statistical analysis was performed on Prism (GraphPad, San Diego, California) using a Mann–Whitney test.

### Assessment of selected DE plasma miRNAs using RT-qPCR

The total RNA was first extracted from plasma using the MagMAX *mir*Vana Total RNA Isolation Kit (Thermo Fisher, Waltman, MA, USA). In addition, an exogenous control C. elegans *miR-39-3p* (1.5 × 10^10^ copies of *cel-miR-39-3p* in 5 µl) was spiked in during the total RNA extraction in order to assess the extraction efficiency, as well as to ensure the inter-plate reproducibility of the RT-qPCR^45,46^. RT-qPCR was performed using the TaqMan Advanced miRNA Assays Kit (Thermo Fisher). A miR-Amp reaction was performed to amplify miRNAs prior to RT-qPCR using the QuantStudio3 (Thermo Fisher). The Cq values of each selected plasma miRNA as well as of two human endogenous control *miR423-5p and miR16-5p*, and of exogenous control *cel-miR39-3p* were measured. The Cq values of the two endogenous controls were compared in order to identify the most stable one to be used to calculate the relative plasma expression^47^:

1 $$\varDelta {{{{{\rm{Cq}}}}}}={{{{{{\rm{Cq}}}}}}}_{{{{{{\rm{miRNA}}}}}}{{{{{\rm{of}}}}}}{{{{{\rm{interest}}}}}}}-{{{{{{\rm{Cq}}}}}}}_{{{{{{\rm{endogenous}}}}}}{{{{{\rm{control}}}}}}}$$

### Three-step Bayesian approach to assess the performance of the multi-omics diagnostic CASH biomarker

A three-step Bayesian approach was implemented using a leave-one-out cross-validation ML framework to assess if a multiomic integrative combination of plasma ratio levels of metabolites and miRNAs enhance the performance of the previously identified protein-based CASH biomarker^25^. This three-step approach was first applied using the plasma ratio levels of (1) metabolites (all: *p* < 0.05), (2) miRNAs, both validated in the independent propensity scored matched validation cohort, and finally (3) the protein-based CASH biomarker as well as the metabolites and miRNAs.

Given the importance of clinical applicability, a smaller number of compounds with high sensitivity was preferred. The accuracy, specificity, sensitivity, receiver operating characteristic curve, and canonical values were computed for each model. The best-performing models were selected by having the highest combination of sensitivity and specificity following the Youden index method^48–50^. A parallel automatic ML framework of correlation-based feature selection (CFS), logistic linear regression with 10-fold cross-validation, and tree-based model selection of the model with the lowest AIC value was used to support these results^51^. Leave-one-out or 10-fold cross-validation ML approaches reduce overfitting effects, while identifying molecules which would individually contribute to the model’s diagnostic association. In addition, AIC criteria allowed for the selection of the least complex model to additionally also prevent overfitting^51^.

The multinomial logistic regression model with a ridge estimator (Weka) was applied as the classifier. This was given a binary classification on ${{{{{\rm{n}}}}}}$ samples with ${{{{{\rm{m}}}}}}$ features. The probability for the positive class for any sample *i* was estimated using

2 $${{P}}\left({{{X}}}_{{{i}}}\right)=\frac{{{{e}}}^{{{{X}}}_{{{i}}}{{\beta }}}}{{{{{{{\rm{e}}}}}}}^{{{{X}}}_{{{i}}}{{\beta }}}+1}$$

where X_i_ is the feature vector for sample ${{{{{\rm{i}}}}}}$ and β is the vector of the ${{{{{\rm{m}}}}}}$ coefficients.

The negative multinomial log-likelihood to be minimized is:

3 $${{L}}=-\mathop{\sum }\limits_{{{i}}=1}^{{{n}}}\left({{{Y}}}_{{{i}}}\right.{{{{{\mathrm{ln}}}}}}\,{{p}}({{{X}}}_{{{i}}})+({1-{{Y}}}_{{{i}}})(1-\,{{{{{\mathrm{ln}}}}}}\,{{p}}({{{X}}}_{{{i}}}))+{{{{{\mathrm{ridge}}}}}} * {{{\beta }}}^{2}$$

where *Y*_*i*_ is the label for sample *i* and ridge = 1.0 × 10^−8^.

A quasi-Newton method was used to search for the optimized values of the ${{{{{\rm{m}}}}}}$ features. Missing values were replaced using mean values. Given the importance of clinical applicability, a smaller number of compounds with high sensitivity was preferred. Therefore, the AIC was calculated to evaluate each possible model using (1) the mean absolute error estimated from logistic regression classification with 10-fold cross-validation, (2) the number of compounds included accounted for the complexity of the model.

### Statistics and reproducibility

PLS-DA was used for the identification of differential metabolites from the unsupervised metabolome (*p* < 0.05, FDR corrected) in the CA (*n* = 53) vs. non-CA (*n* = 17) as well as CASH (*n* = 5) vs. non-CASH (*n* = 48) analyses. Validation of the CASH metabolites in an independent propensity matched cohort of (*n* = 20) CASH and (*n* = 20) non-CASH was also completed using PLS-DA analysis. Unpaired Student’s *t* test was used for the statistical analyses of the canonical values in the CA vs. non-CA as well as CASH vs. non-CASH analyses. The comparisons of the demographics of the discovery and independent propensity score matched cohort were performed with independent samples *t* test, Mann–Whitney *U*-test, χ^2^-test, or Fisher’s exact test using SPSS v22.0 (IBM, Armonk, NY, USA). Continuous variables were tested for normality using Shapiro–Wilk test. All *p*-values were considered statistically significant at α < 0.05.

### Reporting summary

Further information on research design is available in the Nature Portfolio Reporting Summary linked to this article.

## Results

### Correlation of plasma levels of proteins and differentially expressed metabolites

The correlation between the plasma protein levels and normalized metabolite values were assessed using Pearson’s coefficient. A two-sided *t* test was used to estimate the p-value with FDR correction using the R software. The analyses showed that the plasma levels of 5 metabolites and protein were correlated (|*r*| > 0.5; *p* < 0.05, FDR corrected). These include correlations between (1) linoleic acid and C reactive protein (CRP), (2) tauroursodeoxycholic acid and IL-10, (3) glycodeoxycholic acid and vascular endothelial growth factor (VEGF), (4) glycocholic acid and VEGF, as well as (5) Phenylalanylphenylalanine and angiopoietin-1 (ANG-1). These results suggest that these molecules have mechanistic co-involvement. Thereafter, correlational feature selection was used to only include molecules with low inter-correlations and high predictive ability in the diagnostic weighted combinations (Supplementary Fig. 2).

### Transcriptome enriched-KEGG pathways

The transcriptome of human lesional NVUs of CA identified 1542 DEGs (*p* < 0.05, FDR corrected)^36^. The enriched-KEGG pathway analyses were then performed independently on each of the two lists and found 35 enriched-KEGG pathways (*p* < 0.05, FDR corrected; Bayes > 3) for the upregulated DEGs, and 65 (*p* < 0.05, FDR corrected; Bayes > 3) for downregulated DEGs.

### Plasma ratio levels of fifteen metabolites, including cholic acid and hypoxanthine, distinguished CA patients from healthy non-CA subjects

**Fig. 2:**
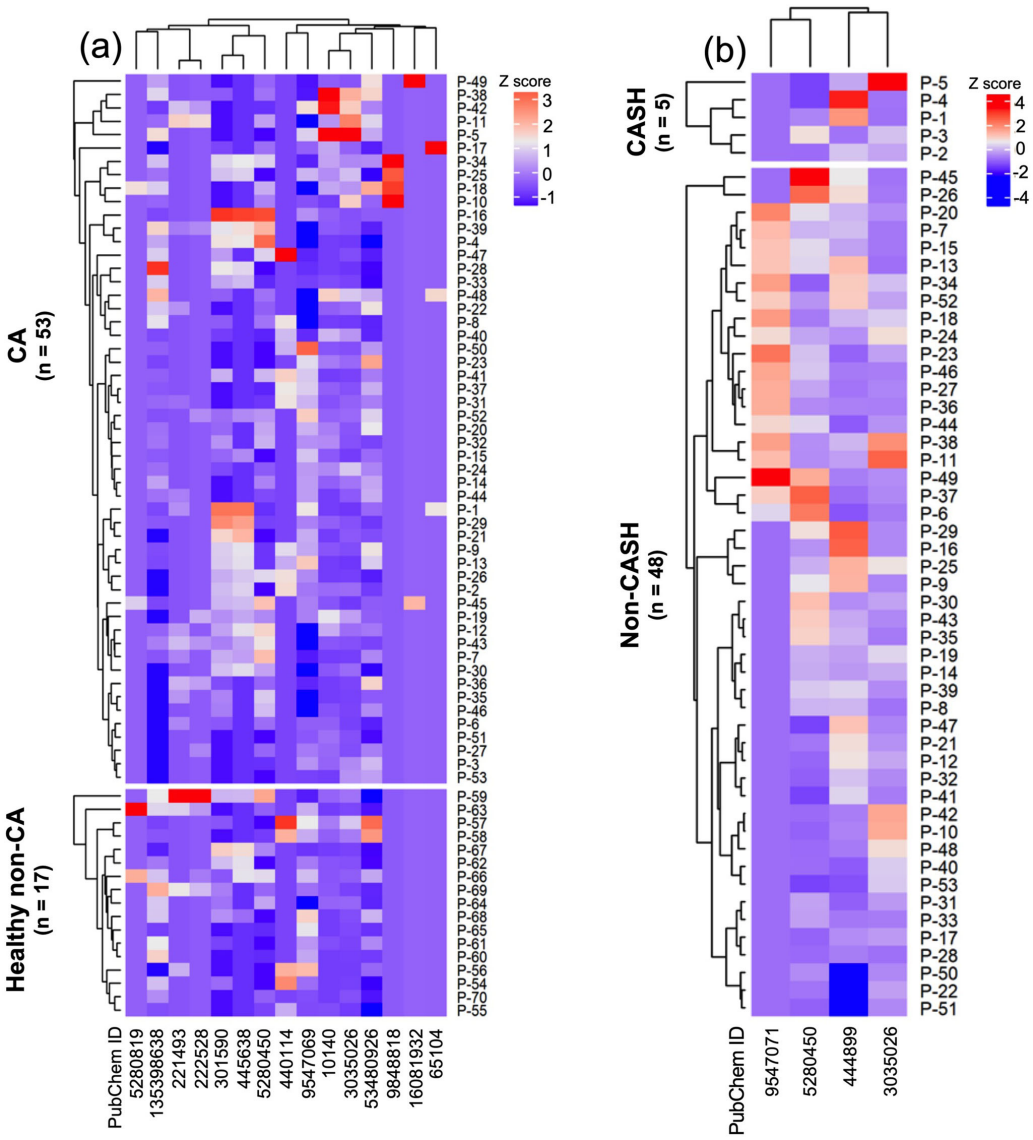
Heatmaps of the differential metabolomic profiles in cavernous angioma (CA) disease and its clinical manifestations. A PLS-Discriminant Analysis showed differences in the plasma levels for (**a**) 15 metabolites between CA patients (*n* = 53 biologically independent patient samples) and healthy non-CA subjects (*n* = 17 biologically independent patient samples), (**b**) four metabolites between cavernous angioma with symptomatic hemorrhage (CASH) patients (*n* = 5 biologically independent patient samples) and cavernous angioma without symptomatic hemorrhage (non-CASH) patients (*n* = 48 biologically independent patient samples) (*p* < 0.05, FDR corrected; Bayes factor>3). Z-scores were calculated to compare metabolite levels across patients within each comparison group, with lower *z* scores (darker blue) representing lower relative plasma levels of metabolites. Metabolites with listed PubChem IDs are identified in Supplementary Tables 2, 3.

**Fig. 3:**
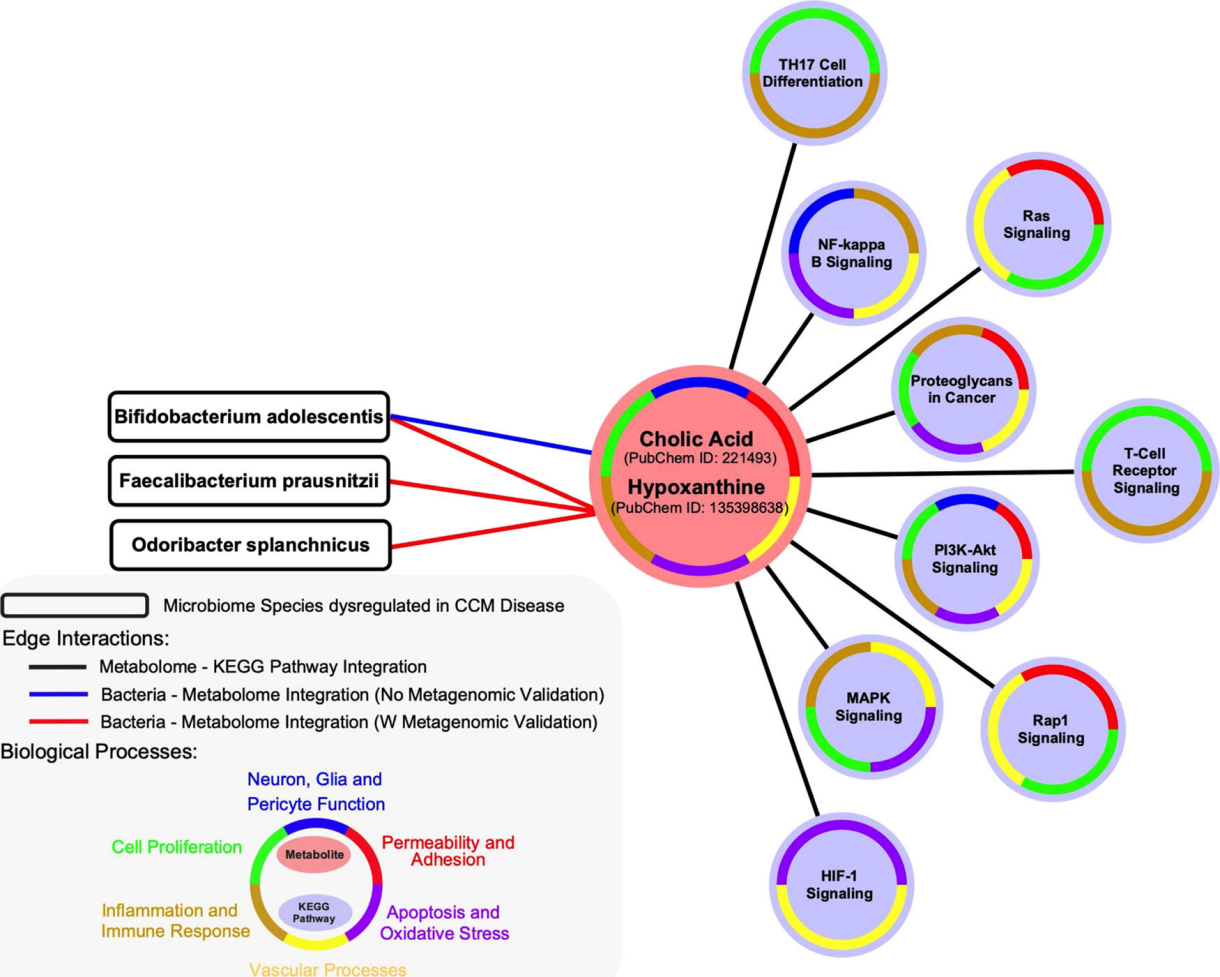
Integrative analyses of cholic acid and hypoxanthine between the differential microbiome, plasma proteome and lesional transcriptome of cavernous angioma (CA) disease. The plasma levels of cholic acid and hypoxanthine, which were lower in CA patients compared to healthy non-CA subjects (*p* < 0.05, FDR corrected), were later linked to *Bifidobacterium adolescentis*, *Faecalibacterium prausnitzii*, and *Odoribacter splanchnicus*. Further integrative analyses identified nine enriched Kyoto Encyclopedia of Genes and Genomes (KEGG)-pathways (*p* < 0.05, false discovery rate [FDR] corrected; Bayes factor>3) that overlap between the differential plasma proteome and metabolome as well as with the lesional transcriptome of CA disease. Reported to have a role in the physiopathogenesis of CAs, these nine pathways were categorized into six biological processes with pre-established relevance to CA disease.

The unsupervised PLS-DA of the metabolome identified 15 molecules with differential plasma ratio levels (*p* < 0.05, false discovery rate [FDR] corrected) in a discovery cohort comprised of 53 consecutive CA patients enrolled between April 2017 and August 2018 compared to 17 healthy non-CA controls (Fig. 2a, Supplementary Table 1, Supplementary Table 2, Supplementary Data 3). This discovery cohort contains the same patients reported in our recently published study on permissive microbiome in CA disease^17^. In silico integrative analyses with the microbiome bacterial species that had previously been shown in different abundance (*p* < 0.01, FDR corrected) among CA vs healthy non-CA (i.e., 16S-RNA seq) revealed interactions of cholic acid and hypoxanthine with genes of *Bifidobacterium adolescentis*, *Faecalibacterium prausnitzii*, and *Odoribacter splanchnicus* (Fig. 3, Supplementary Data 4)^17^. Of interest, ten genes linking the three species to hypoxanthine were identified within shotgun metagenomic sequencing of the differential microbiome of the same CA versus non-CA subjects^17^.

Further in silico analyses showed that nine of the 15 differential metabolites were involved in 20 KEGG pathways commonly enriched within the previously published transcriptome of human lesional NVUs^36^ and differential proteome of CA disease (*p* < 0.05, FDR corrected; Bayes factor>3; Fig. 3, Supplementary Fig. 3, Supplementary Data 4). Of interest, cholic acid and hypoxanthine were related to PI3K-AKT, MAPK, NF-KB, and Rap1 signaling (Fig. 3, Supplementary Data 4, Supplementary Data 5) previously implicated the pathogenesis of CAs^16,20–22,52,53^.

The individual receiver operating characteristic (ROC) curves for each of the fifteen metabolites showed poor or fair sensitivity/specificity to distinguish CA and non-CA healthy subjects (Supplementary Table 2). A three step Bayesian ML approach, however, further derived a weighted combination of five differential plasma metabolites able to distinguish CA and non-CA healthy subjects of the discovery cohort with 87.5% sensitivity and 89% specificity:

4 $$\begin{array}{c}{{{{{\rm{Diagnostic}}}}}}\,{{{{{\rm{CA}}}}}}\,{{{{{\rm{biomarker}}}}}}=858.65\ast [{{{{{\rm{linoleic}}}}}}\,{{{{{\rm{acid}}}}}}]+\,1005.39\ast [{{{{{\rm{glycocholic}}}}}}\,{{{{{\rm{acid}}}}}}]\\ +1091.07\ast [{{{{{\rm{hydroxypalmitic}}}}}}\,{{{{{\rm{acid}}}}}}]-1857.99\ast [{{{{{\rm{urobilin}}}}}}]\\ -2145.29\ast [7{{{{{\rm{a}}}}}}-{{{{{\rm{hydroxy}}}}}}-3-{{{{{\rm{oxo}}}}}}-4-{{{{{\rm{cholestenoic}}}}}}\,{{{{{\rm{acid}}}}}}]+0.07\end{array}$$

Canonical values estimated with this model were higher in CA patients compared to healthy non-CA patients (*p* < 0.0001). This sensitivity and specificity of the combined metabolites in differentiating CA from non-CA subjects is similar to that reported with the permissive microbiome in the same subjects^17^. Plasma metabolites reflecting the permissive microbiome and other mechanisms distinguishing CA from non-CA subjects are much more amenable to assays in individual subjects than analyses of fecal bacterial composition.

### The plasma ratio levels of four metabolites, including linoleic and arachidonic acids, distinguished CASH and cavernous angioma without symptomatic hemorrhage (non-CASH) patients

**Fig. 4:**
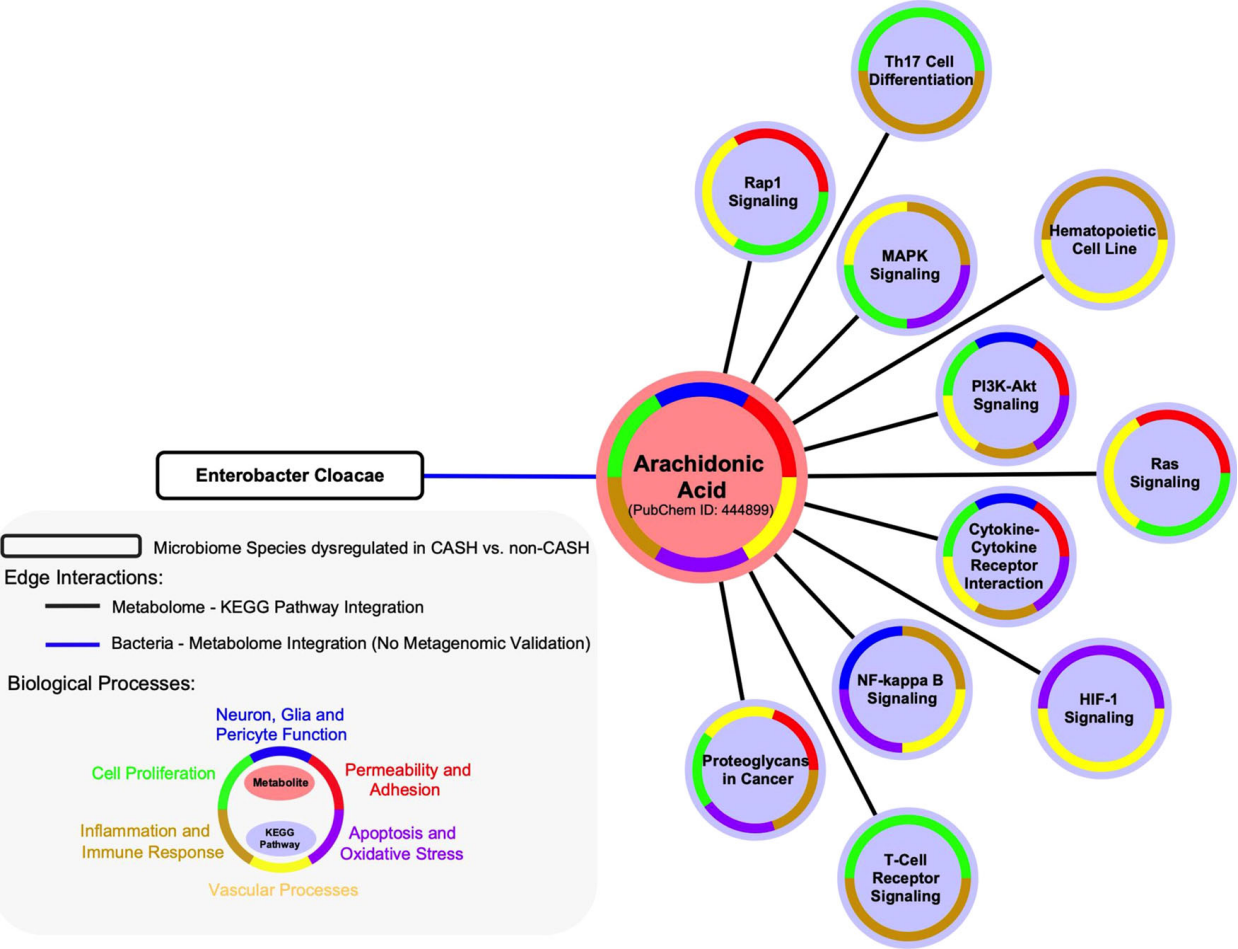
Arachidonic acid links *Enterobacter cloacae* and 11 Kyoto Encyclopedia of Genes and Genomes (KEGG) pathways enriched across the differential proteome and transcriptome of cavernous angioma with symptomatic hemorrhage (CASH) patients. The integrative analyses draw a link between higher plasma levels of arachidonic acid and *Enterobacter cloacae* in CASH patients (*p* < 0.05, false discovery rate [FDR] corrected; Bayes factor>3). Further analyses also identified 11 enriched-KEGG pathways with a role in cavernous angioma (CA) disease that overlapped between the differential plasma metabolome and proteome as well as the lesional transcriptome of CA disease. These 11 pathways were then categorized into six biological processes mechanistically related to CA disease (*p* < 0.05, FDR corrected; Bayes factor>3).

The results of the unsupervised PLS-DA identified showed that the plasma ratio levels of linoleic and arachidonic acids were greater in CASH (*n* = 5) compared to non-CASH (*n* = 48) patients in the discovery cohort, and lower for 1-oleoyl-sn-glycero-3-phosphoethanolamine and glycodeoxycholic acid (all: *p* < 0.05, FDR corrected; Fig. 2b, Supplementary Table 3A, Supplementary Data 3). Arachidonic acid in fact interacts with specific genes of *Enterobacter cloacae*, which has been found to be differentially abundant in CASH compared to non-CASH patients^17^. Further in silico analyses showed that arachidonic, linoleic, and glycodeoxycholic acids were mapped through 23 common KEGG pathways across the CA transcriptome and proteome (Supplementary Fig. 4, Supplementary Data 5), including PI3K-Akt, MAPK, HIF-1, and Rap1 signaling. Of interest, arachidonic acid was specifically correlated with 11 enriched-KEGG pathways common across the transcriptome, proteome, and metabolome datasets (*p* < 0.05, FDR corrected; Bayes factor>3; Fig. 4, Supplementary Data 4, Supplementary Data 5).

### The four differentially present plasma metabolites identified in CASH patients, compared to non-CASH, were validated in an independent propensity matched cohort

A CASH is a cardinal event in the clinical course of CA disease^14^. An independent propensity matched cohort of CASH (*n* = 20) and non-CASH (*n* = 20) patients identified among subsequent prospectively enrolled subjects, was used to validate the metabolomic findings (Supplementary Table 4). Twenty CASH and 20 non-CASH patients were propensity score matched. The supervised PLS-DA also showed that plasma ratio levels of linoleic (Supplementary Fig. 1) (*p* = 0.003) and arachidonic (*p* = 0.06) acids were greater in CASH compared to non-CASH patients of the validation cohort, and lower for 1-oleoyl-sn-glycero-3-phosphoethanolamine (*p* = 0.02) and glycodeoxycholic acid (*p* = 0.0002) in CASH compared to non-CASH patients, as in the discovery cohort, and in the same direction (Supplementary Fig. 5**;** Supplementary Table 3B).

### The plasma ratio levels of six metabolites, including bilirubin, distinguished aggressive vs. non-aggressive CA patients

Aggressivity of CA disease is an important determinant for understanding CA clinical course and developing therapeutic strategies. Among the 53 CA patients of the discovery cohort, 19 were classified into aggressive and 34 as non-aggressive disease based on their clinical records. Aggressive CA disease was defined as: (1) age of symptom onset prior to 18 years of age, (2) a history of two or more CASH events, (3) greater than 25 susceptibility weighted imaging (SWI) lesions, or (4) greater than 5 T_2_-weighted lesions >4 mm in diameter^10,17,23^. The differential metabolome between aggressive and non-aggressive disease CA patients included in the discovery cohort identified 6 metabolites (*p* < 0.05, FDR corrected; Supplementary Fig. 6A, Supplementary Table 5) in the discovery cohort. Among these metabolites, bilirubin, as well as cis-9-hexadecenoic and tauroursodeoxycholic acids interacted with 19 CA-enriched-KEGG pathways that also overlapped across the transcriptome and proteome of CA disease (Supplementary Data 5). Of these 19, the literature showed 10 to be KEGG pathways with prior established relevance to CA disease (Supplementary Fig. 7, Supplementary Data 5).

### Three metabolites, including glycodeoxycholic acid and glycocholic acid, distinguished familial- vs. sporadic-CA patients

CA patients including within the discovery cohort harboring (1) multiple lesions throughout the brain on T_2_-weighted imaging or SWI, (2) a documented *CCM1*, *CCM2*, or *CCM3* germline mutation, and/or (3) first-degree relative with a history of CA were classified as familial-CA (*n* = 25). Patients showing a solitary or a cluster of lesions associated with a developmental venous anomaly lesion were identified as sporadic-CA patients (*n* = 28)^2^. The plasma levels of glycodeoxycholic and glycocholic acids, as well as decanoyl-L-carnitine were different (*p* < 0.05, FDR corrected; Supplementary Fig. 6B, Supplementary Table 6) between familial- and sporadic-CA patients of the discovery cohort. Two hundred thirty-one pathways were associated with the genes that interact with or are affected by these three metabolites. Among these 231 pathways, 37 overlapped with the enriched-KEGG pathways identified in the human lesional NVUs transcriptome. Of the 37 enriched-KEGG pathways, 12 also overlap with the proteome. Seven of these pathways had CA relevance from the literature, including PI3K-Akt, HIF-1, and MAPK signaling (Supplementary Fig. 8, Supplementary Data 5).

### Nineteen plasma miRNAs are differentially expressed in the propensity matched cohort of 20 CASH and 20 non-CASH patients

Differential expression analyses identified 19 DE plasma miRNAs (*p* < 0.05, FDR corrected) between the CASH and non-CASH patients of the propensity matched cohort (Supplementary Data 6). Among these 19 DE miRNAs, 8 were assessed using RT-qPCR. Plasma DE *miR-486-5p*, *miR-25-3p*, *miR-16-5p, miR-183-5p* and *miR-501-3p* were independently discovered in previous preliminary analyses^25^. In addition, *miR-182-5p*, *miR-20a-5p* and *miR-92a-3p* had at least 2 putative gene targets within the transcriptome of lesional human CA NVUs^25,36^, related to enriched-KEGG pathways with documented mechanistic role in the physiopathogenesis of CAs including HIF-1, MAPK, PI3K-Akt, Rap1 and VEGF signaling^20–22,54^.

### Diagnosis of CA characteristics with metabolites alone

*Weighted combination to distinguish aggressive and non-aggressive CA patients*. The weighted model to distinguish patients with aggressive and non-aggressive disease of the discovery cohort achieved 85% sensitivity and 79% specificity:

5 $$\begin{array}{c}L({{{{{\rm{non}}}}}}{\mbox{-}}{{{{{\rm{aggressive}}}}}}\,{{{{{\rm{disease}}}}}})= 3682.02 \ast [{{cis}}{\mbox{-}}9{\mbox{-}}{{{{{\rm{hexadecenoic}}}}}}\,{{{{{\rm{acid}}}}}}] \\ -19165.94 \ast [1{\mbox{-}}{{{{{\rm{palmitoyl}}}}}}{\mbox{-}}{{{{{\rm{sn}}}}}}{\mbox{-}}{{{{{\rm{glycero}}}}}}{\mbox{-}}3{\mbox{-}}{{{{{\rm{phosphocholine}}}}}}]\\ +962.10 \ast [{{{{{\rm{bilirubin}}}}}}]{\mbox{-}}0.48\end{array}$$

The canonical values derived from this model were lower in CA patients with aggressive disease (*p* < 0.0001).

#### Weighted combination to distinguish familial- and sporadic- CA patients

Finally, the best weighted model, including only the plasma metabolites differentially expressed between the familial-CA and sporadic-CA patients of the discovery cohort, achieved 37% sensitivity and 86% specificity.

6 $$L({{{{{\rm{Familial}}}}}}{\mbox{-}}{{{{{\rm{CA}}}}}})=1236.91\ast [{{{{{\rm{decanoyl}}}}}}{\mbox{-}}{{{{{\rm{L}}}}}}{\mbox{-}}{{{{{\rm{carnitine}}}}}}]{\mbox{-}}0.37$$

The canonical values derived from this last analysis trended higher in familial-CA disease compared to sporadic-CA disease (*p* = 0.09).

### Multi-omics integration of plasma levels of metabolites, miRNAs and proteins enhances the diagnostic association with CASH

Levels of DE miRNAs had not been previously considered in combined biomarker analyses. We hence sought to examine whether the combination of previously identified proteins and miRNAs differentiating CASH from non-CASH along with the differential metabolites can enhance the sensitivity and specificity of the diagnostic association.

**Fig. 5:**
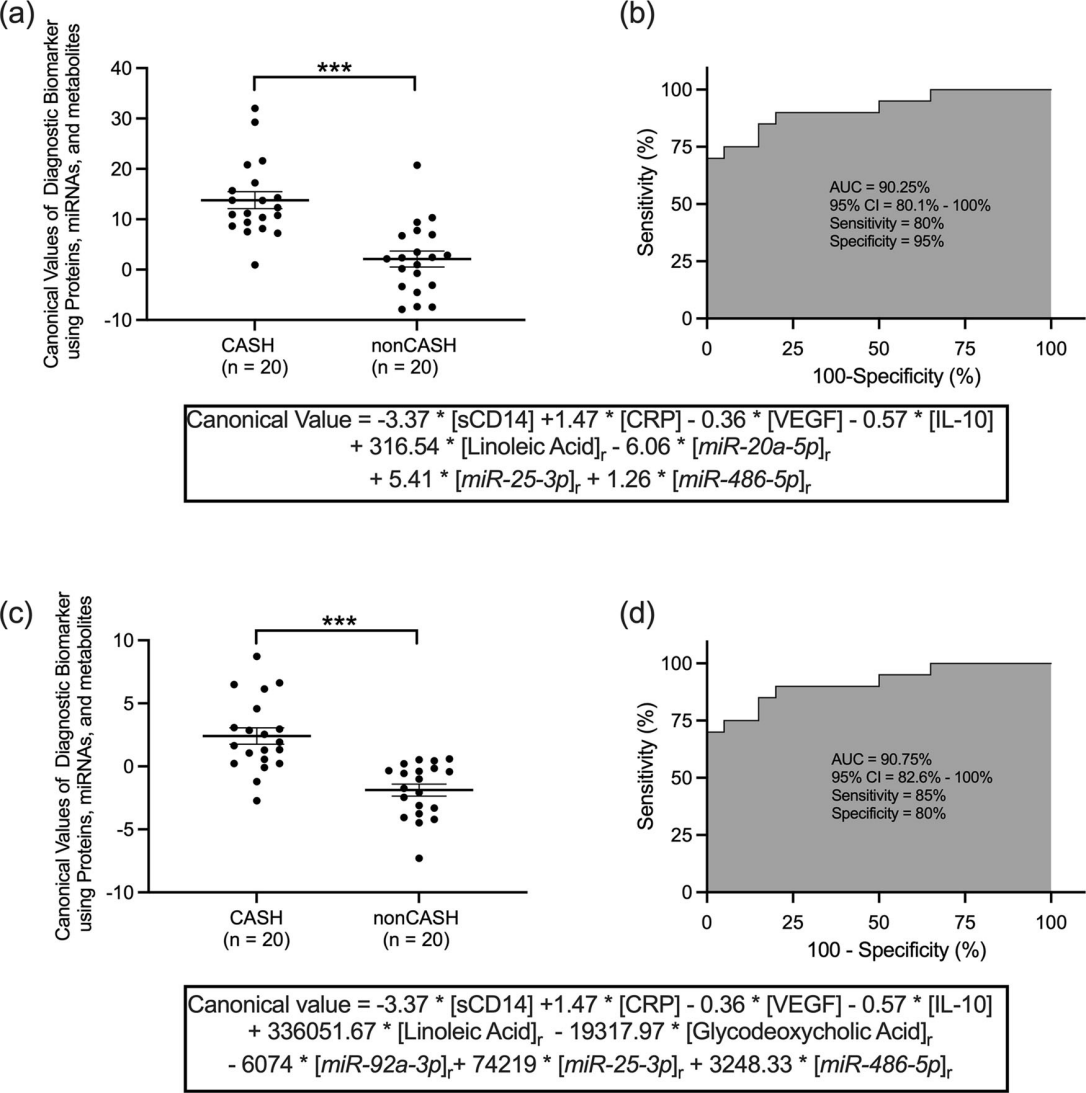
Weighted combinations of plasma ratio of metabolites and micro-RNAs (miRNAs) improve the performance of the proteins-based cavernous angioma with symptomatic hemorrhage (CASH) biomarker. The canonical values estimated with the optimal integrative weighted model were (**a**) higher (unpaired Student’s *t* test, *p* < 0.0001) in the CASH (*n* = 20 biologically independent patient samples) compared with cavernous angioma without symptomatic hemorrhage (non-CASH) patients (*n* = 20 biologically independent patient samples) included in the independent propensity matched validation cohort. **b** The receiver operating characteristic (ROC) analyses showed that this integrative model differentiated CASH patients from non-CASH patients with 80% sensitivity and 95% specificity (area under the curve [AUC] = 90.25%). **c** An alternative optimal integrative weighted model developed using a parallel 10-fold cross-validation machine learning approach showed higher canonical values higher (unpaired Student’s *t* test*, p* < 0.0001) in CASH compared with non-CASH patients. **d** The ROC analysis showed that the alternative optimal integrative model differentiated CASH from non-CASH with 85% sensitivity and 80% specificity (AUC = 90.75%). CI, confidence interval; ***p < 0.001. Error bars re*p*resent standard error of the mean.

First, the previously published weighted combination of four proteins was again confirmed to distinguish CASH and non-CASH patients of the independent propensity score matched cohort with 60% sensitivity and 70% specificity (Accuracy=69.3%, area under the curve [AUC] = 69.2%), in the same range as reported previously in a different cohort^25^. A three steps Bayesian approach using a leave-one-out cross-validation (CV) ML framework was then implemented to determine if a weighted combination of plasma levels of metabolites, proteins and miRNAs improves the diagnostic association with CASH (Supplementary Fig. 9**)**. The integrative weighted combination of plasma levels of one metabolite, three miRNAs and the same protein CASH biomarkers improved the diagnostic association distinguishing CASH patients versus non-CASH patients to 80% sensitivity and 95% specificity (Accuracy = 87.5%, AUC = 90.3%, Akaike Information Criterion [AIC] = 45.6; Fig. 5, Supplementary Data 7):

7 $$\begin{array}{c}{{{{{\rm{Integrated}}}}}}\,{{{{{\rm{diagnostic}}}}}}\,{{{{{\rm{CASH}}}}}}\,{{{{{\rm{biomarker}}}}}}=-3.37 * [{{{{{\rm{sCD}}}}}}14]+1.47\ast [{{{{{\rm{CRP}}}}}}]\\ -0.36\ast [{{{{{\rm{VEGF}}}}}}]-0.57\ast [{{{{{\rm{IL}}}}}}-10]+316.54\ast {[{{{{{\rm{linoleic}}}}}}{{{{{\rm{acid}}}}}}]}_{{{{{{\rm{r}}}}}}}\\ -6.06\ast {[miR-20a-5p]}_{{{{{{\rm{r}}}}}}}+5.41\ast {[miR-25-3p]}_{{{{{{\rm{r}}}}}}}+1.26\ast {[miR-486-5p]}_{{{{{{\rm{r}}}}}}}\end{array}$$

The canonical values estimated using this integrated diagnostic CASH biomarker were higher in CASH compared to non-CASH patients (*p* < 0.0001; Fig. 5, Supplementary Data 7).

A parallel 10-fold cross-validation ML approach yielded similar results with 85% sensitivity and 80% specificity (Accuracy = 82.5%, AUC = 90.8%, AIC = 31.9).

## Discussion

We performed unsupervised differential plasma metabolomic analysis in CA patients versus healthy non-CA subjects, and in CA patients with different disease features. Multiomic integrative analyses showed that several differential metabolites were associated not only with enriched-KEGG pathways dysregulated within the transcriptome of lesional NVUs and plasma proteome, but also with gut microbiota species showing differential relative abundances in CA patients. This is the first plasma metabolomic analysis in any neurovascular disease. Metabolites with different levels in CAs with and without SH, the most important clinical feature of the disease, were validated in a propensity matched independent patient cohort. A Bayesian approach implemented with ML algorithms showed that normalized levels of plasma metabolites and miRNAs enhanced the performance of a previously published protein-based CASH biomarker. This integrative biomarker development strategy is applicable in other pathologies.

The initial differential analyses of the metabolome showed that the plasma concentrations of cholic, glycocholic, glycodeoxycholic, and tauroursodeoxycholic acids were different between CA patients and healthy non-CA subjects. Cholic and glycocholic acids are two compounds synthesized in the liver and, after conjugation with glycine, secreted in bile^55^. These molecules have previously been shown to play a role in maintaining blood–brain barrier integrity, downregulating apoptosis, and mitigating inflammatory damage following a hypoxic state^56^. Of interest, the plasma levels of glycocholic acid and VEGF were correlated (Supplementary Fig. 2). It has been shown that glycocholic acid inhibits VEGF-induced angiogenesis in choroidal endothelial cells, and protects against oxidative damage^57^. Recently, TLR4-MEKK3-KLF2/4 signaling, driven by the gut microbiome via gram-negative bacterial lipopolysaccharide, has been postulated to drive CA physiopathogenesis^16^. Specifically, this study suggested that TLR4, in association with its co-receptor CD14 on brain endothelial cells, stimulates MEKK3-KLF2/4 signaling^16^. Specific to CA disease, activated MEKK3-KLF4 signaling could impair PI3K-mTOR signaling, while also increasing expression of a second MEKK3 effector, the secreted versicanase ADAMTS5, increasing CA lesion formation^58^.

While mutations in the CCM proteins have noteworthy structural effects on endothelial cells, previous studies have also shown an inflammatory component in CA disease. Recent studies have shown oligoclonal IgG synthesis, immune complex formation, and inflammatory cell infiltration, including CD4^+^ and CD8^+^ T-cells, within CA lesions^59^. The relationship of cholic acid and hypoxanthine with several inflammation-related KEGG pathways is consistent with their association with CA lesion development, and their potential role as disease biomarkers.

It is well-documented that the risk of re-bleeding after a first CASH increases approximately 10-fold, however, it is unclear what predisposes these patients to this increased susceptibility^11^. Ongoing inflammation and angiogenesis may contribute to this underlying process. Further differential analyses of the metabolome showed greater plasma levels of arachidonic and linoleic acids in CASH patients. The in silico incorporation of microbiota data showed that arachidonic acid identified a link to *Enterobacter cloacae*, while also interacting with MAPK/MEKK3/ERK3, PI3K-Akt, Rap1, and T-cell receptor signaling pathways. Cyclooxygenase-1 (COX-1) and cyclooxygenase-2 (COX-2) downstream synthesized products of arachidonic acid, play a role in mediating angiogenesis as well as inflammatory interactions of leukotrienes and prostaglandins^60^. We note that LPS, in addition to its role in TLR4 signaling and MAPK/MEKK3/ERK3 upregulation, induces COX-1 and COX-2 expression, possibly explaining the increased arachidonic acid seen in CASH patients^61^. In addition, linoleic acid was shown to interact with the NF-KB pathway, which is important in the regulation of both the adaptive and innate immune systems as well as acting as a mediator of inflammatory response^62^. Activation of this pathway has been associated with inhibition of angiogenesis^62^.

The plasma levels of the vast majority of metabolites and putative proteins previously associated with CA disease^23–25^ did not correlate, and hence contributed complementary biomarker information (Supplementary Fig. 2). Consistent with this observation, the three step Bayesian approach using a ML framework showed that multiomic integration improves the efficacy of CASH diagnosis, the most relevant clinical context in this disease. The molecules embedded in the best weighted biomarker combination have strong mechanistic rationale in CA disease.

Lower plasma concentrations of bilirubin were observed in CA patients experiencing a more aggressive clinical course during their lifetime. Bilirubin is a catabolic product of heme from the hepatic reticuloendothelial system that has antioxidant properties protecting the NVUs^63,64^. It may be cautiously interpreted that decreased levels of bilirubin may also alter angiogenic signaling through *VEGFR1* as well as play a role in altering MAPK/MEKK3/ERK3 signaling, therefore increasing lesion number, formation, and hemorrhagic activity^65^. In addition, lower plasma levels of bilirubin have been reported in cerebrovascular diseases, such as deep white matter hyperintensities^66^. Oxidative stress related to low bilirubin levels is consistent with the pro-inflammatory characteristics observed in-situ in CAs^66^. Lower levels of bilirubin in patients with aggressive CA disease suggest the first possible link between reduced bilirubin antioxidant effect and cumulative activity of CA disease during a patient’s lifetime, which will need to be confirmed in mechanistic studies.

The genotype analyses differentiating familial- versus sporadic-CA patients revealed many KEGG pathways central to CA disease such as those mentioned above. However, given the low accuracy, and high mean absolute error, even the combination of plasma metabolites and proteins was unable to develop an accurate biomarker of genotype. There were small numbers of cases with each genotype, and larger cohorts might improve the performance of combinations of plasma metabolites and proteins. The incorporation of other circulating molecules, such as miRNAs may increase the accuracy and develop a more clinically applicable diagnostic biomarker of familial disease^25^.

This is a single site study, with inherent limitations and potential biases of referrals and enrollments. The imbalance between the number of CASH and non-CASH cases in the discovery cohort reflects the natural clinical course of this disease, with fewer than 15% of cases seen in clinical practice representing CASH^11^. The discovery cohorts were not matched for sex, age, disease features, or other potential confounders, and the small sample did not allow for specific analyses of interactions between metabolites and these features. However, the results of the discovery cohort were further validated in a four-fold larger independent cohort of CASH cases, propensity matched for potential confounders (age, sex, brainstem lesion location, and phenotype)^13^ with an identical number of non-CASH cases. The numbers of cases enrolled in discovery and validation cohorts are the largest ever reported in this rare disease, providing a first proof of concept for combined plasma metabolite, protein, and miRNA biomarkers for improved accuracy in specific clinical contexts of use. Because of these results, metabolomic discovery will now be incorporated in an ongoing large study sponsored by the U.S. National Institutes of Health (R01 NS114552) aimed at developing biomarkers of CASH, with a sample size powered to examine independent effects of multisite enrollment, age, sex, lesion location and genotype^13^.

Another limitation is that the lesional transcriptome data^36^ was not from the same individuals as the microbiome, metabolome and proteome. The lesional transcriptome can only be derived from patients undergoing surgical lesion resection. Previously published microbiome data included the discovery cohort reported herein where metabolites were identified^17^.

Our protein assays included candidate molecules implicated in CA disease^23–25^. It is possible that other proteins might emerge as promising targets in unsupervised proteomic analyses, or in future mechanistic studies. We tested herein candidate protein biomarkers of CASH that had shown significant associations in previous studies with independent cohorts.

The metabolomic discoveries were unsupervised, and included peaks related to known drug molecules and their metabolites (e.g., such as anticonvulsants, migraine medications) commonly used in CA patients. These were discarded. However, several potentially meaningful molecules in the differential metabolome were unannotated and could not be identified by current techniques, and these may contribute mechanistic links or strong biomarker performance not examined herein. In addition, there are limitations to mass spectrometry with respect to isomer differentiation. We mitigated this concern by using internal standards of the compounds from our discovery study^67,68^. Differentiation of all isomers can be performed using nuclear magnetic resonance or high-performance liquid-chromatography techniques but is outside the scope of this work^69^. Many of the identified metabolites were remarkably linked to relevant CA lesion transcriptome, disease microbiome, and mechanistically plausible pathways. While these molecules may play important roles as biomarkers of CA disease, currently, mechanistic conclusions cannot be made about the causal relationships of these molecules to CA pathogenesis. Further mechanistic understanding of their roles in disease pathology could lead to the development of therapeutics in the treatment of CA disease. Following establishment of diagnostic biomarkers, studies shall be undertaken to develop prognostic biomarkers to predict future symptomatic hemorrhage as well as monitoring biomarkers to monitor the effects of therapies^70^.

Plasma levels of metabolites reflecting the permissive gut microbiome are easier to assay in the clinical context, than the analysis of bacterial genes in fecal specimens. Circulating levels (assayed by qPCR) of miRNAs that had been shown to be DE in discovery cohorts, also present major advantages in comparison to the sequencing of plasma microRNome in individual subjects.

We identify interactions of the differential plasma metabolome and proteome, lesion transcriptome, and gut microbiome associated with CA, resulting in cross-validation of postulated mechanisms of disease. Bayesian and ML algorithms implemented herein led to the development of highly accurate markers of CA diagnosis and CASH. The levels of mechanistically linked metabolites, miRNAs and proteins were validated in independent cohorts, and their weighted combination enhanced biomarker performance in relation to SH, the most relevant clinical context of use. The testing of these biomarkers in larger populations across multiple sites could lead to further confidence in smart blood tests based on multiomic mechanistic links in this and other diseases.

## Supplementary information

Supplemental Information

Supplemental Data 1

Supplemental Data 2

Supplemental Data 3

Supplemental Data 4

Supplemental Data 5

Supplemental Data 6

Supplemental Data 7

Description of Additional Supplementary Files

Reporting Summary

## Data Availability

All metabolomic data for the discovery cohort study are available in the GNPS repository, at https://gnps.ucsd.edu/ProteoSAFe/status.jsp?task=b82b664f25854df492aeec5420b95d45. All metabolomic data for the validation cohort study are available on the MassIVE repository, Accession number MSV000091098, https://massive.ucsd.edu/ProteoSAFe/dataset.jsp?task=d833d707661e432d91dc91c67d76d1ee. Data underlying all figures in the main manuscript are provided as supplementary data files. Supplementary Data 3 contains the source data for Fig. 2. Supplementary Data 4 contains the source data for Figs. 3, 4. Supplementary Data 7 contains the source data for Fig. 5. Any additional records are available from the corresponding authors upon reasonable request.

## Code availability

All scripts used in the generation of this data can be found at 10.5281/zenodo.7630546^71^.
