# Supplementary material for: Plasma metabolites with mechanistic and clinical links to the neurovascular disease cavernous angioma
Source: Commun Med (Lond). 2023 Mar 3;3:35. doi: 10.1038/s43856-023-00265-1 (PMC9984539; doi:10.1038/s43856-023-00265-1)
Supplement: Supplementary file 1 — Supplemental Information [file 43856_2023_265_MOESM1_ESM.pdf]

Supplemental Information

Plasma metabolites with mechanistic and clinical links to the neurovascular disease cavernous angioma

Abhinav Srinath[1,†]; Bingqing Xie[2,†]; Ying Li[1,3]; Je Yeong Sone[1]; Sharbel Romanos[1]; Chang Chen[4], Anukriti Sharma[5,6]; Sean Polster[1]; Pieter C. Dorrestein[6,7]; Kelly C. Weldon[6]; Dorothy DeBiasse[1]; Thomas Moore[1]; Rhonda Lightle[1]; Janne Koskimäki[1]; Dongdong Zhang[1]; Agnieszka Stadnik[1]; Kristina Piedad[1]; Matt Hagan[1]; Abdallah Shkoukani[1]; Julián Carrión-Penagos[1]; Dehua Bi[8]; Le Shen[1,5]; Robert Shenkar[1]; Yuan Ji[8]; Ashley Sidebottom[9]; Eric Pamer[9]; Jack A. Gilbert[5,6], Mark L. Kahn[10]; Mark D'Souza[9]; Dinanath Sulakhe[9]; Issam A. Awad[1,*,#] ; Romuald Girard[1,#]

*Corresponding author
[†]These authors contributed equally
[#]These authors jointly supervised this work

Supplemental Figures

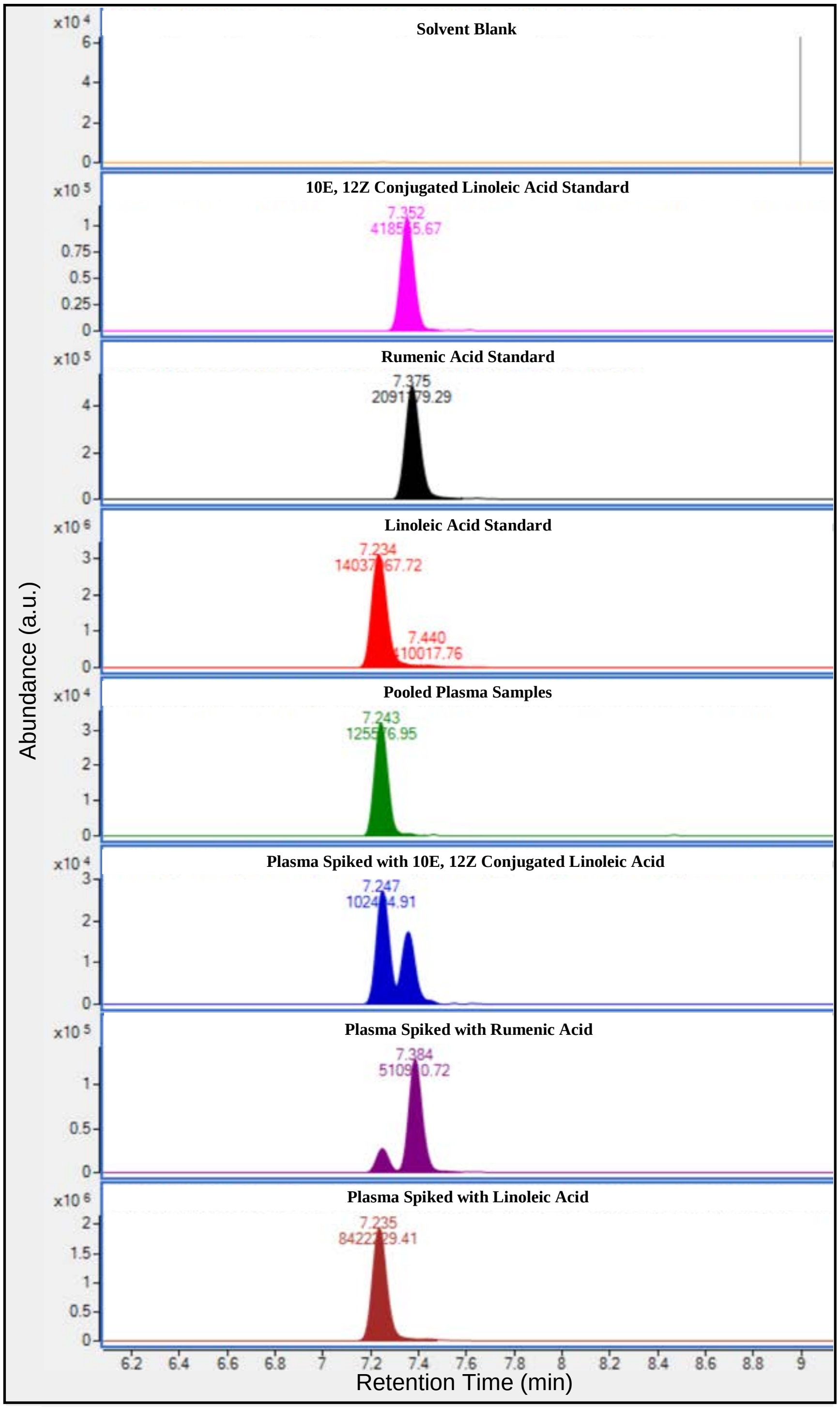

Supp. Fig. 1. Level 1 Confirmation of Linoleic Acid. A level 1 confirmation was performed to support the results of linoleic acid using predefined standards for 10E, 12Z-Conjugated Linoleic; Rumenic; and Linoleic acids as well as plasma samples. The results confirmed that linoleic acid shared the exact fragmentation profile, m/z ratio, and retention time with the molecule identified in the pooled patient samples.

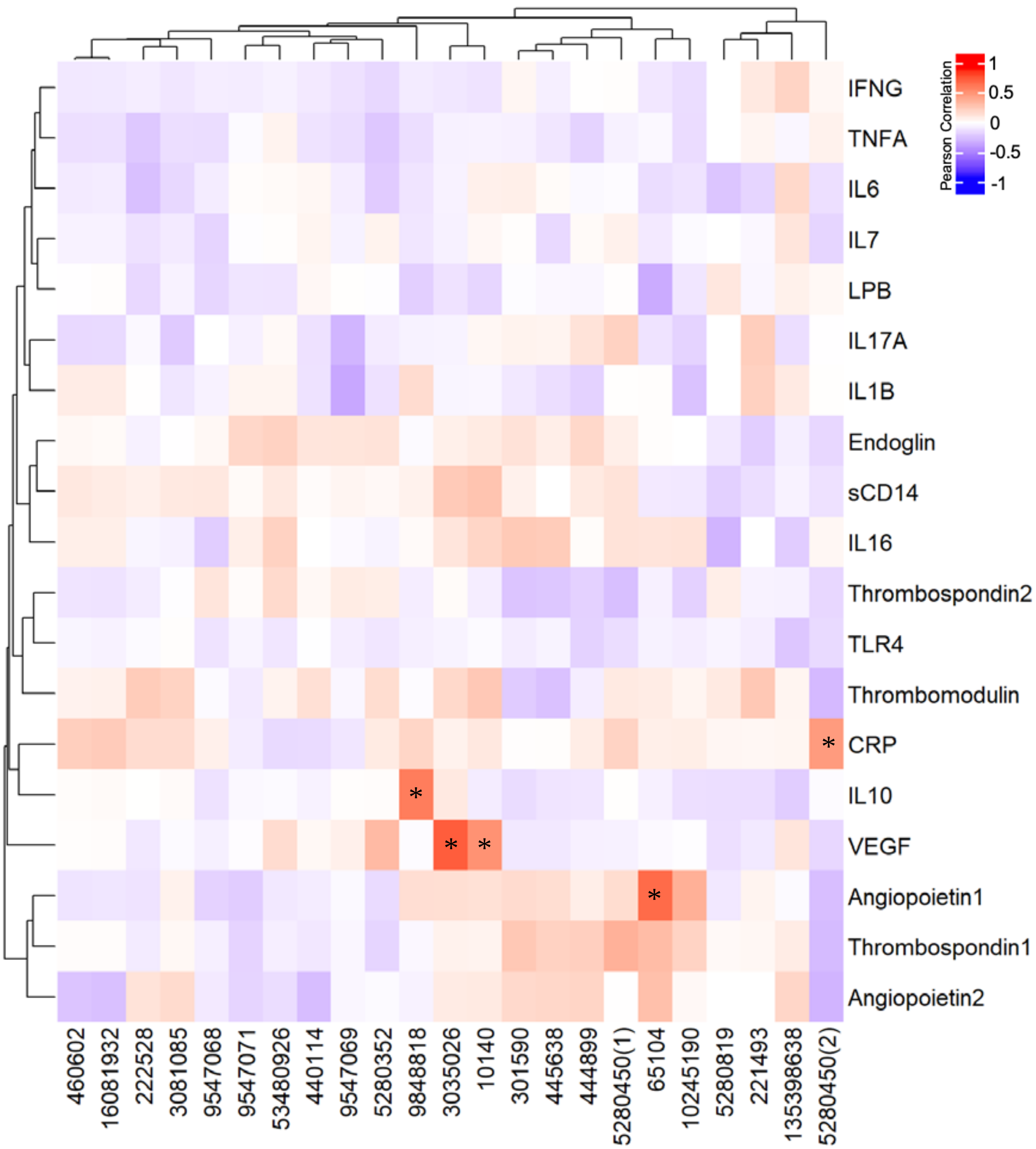

Supp. Fig. 2. Heatmap showing the correlation between plasma protein levels and differential metabolites. Plasma levels of the 19 proteins were correlated with normalized plasma levels of 23 metabolites using Pearson coefficient. The plasma levels of five metabolites were correlated with the plasma levels of five proteins and could implicate biological mechanistic relationships between the molecules. Pubchem ID compound names can be found in **Supp. Tables 2, 3, 5, 6**. *; p<0.05, false discovery rate corrected.

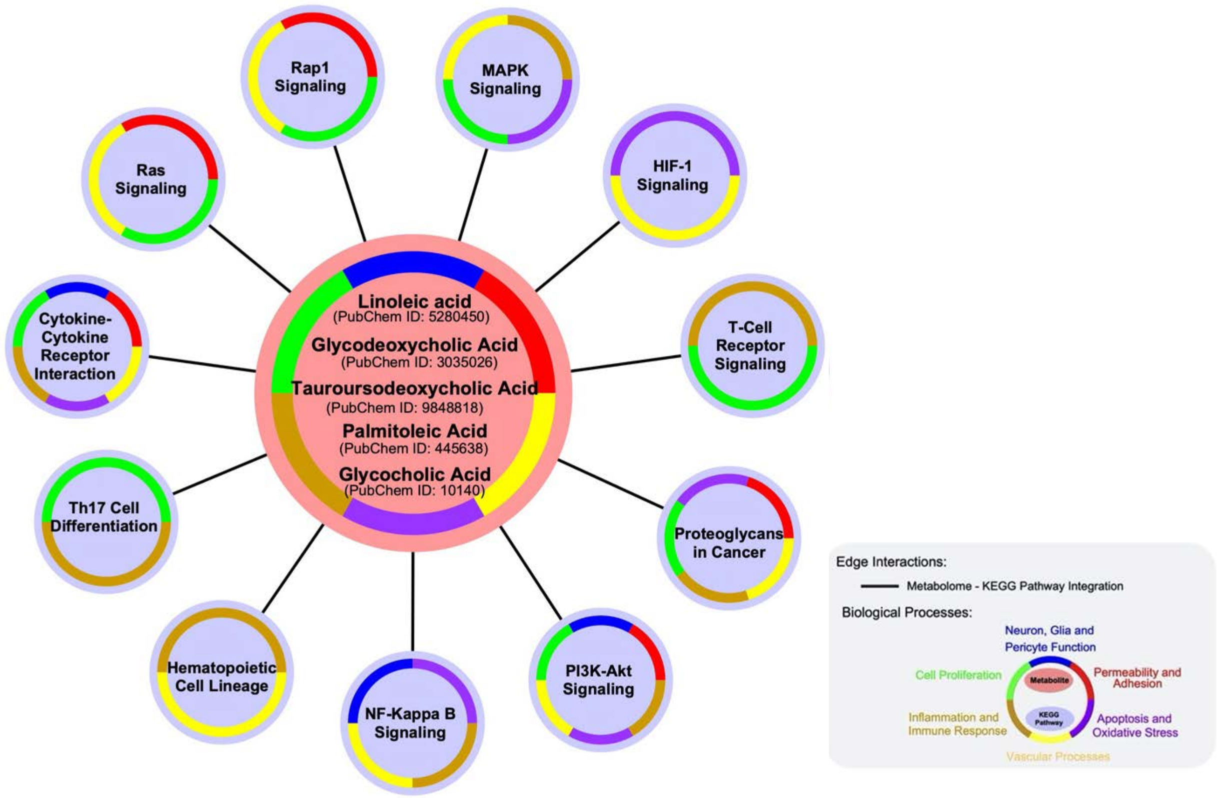

Supp. Fig. 3. Eleven enriched- Kyoto Encyclopedia of Genes and Genomes (KEGG) pathways identified in cavernous angioma (CA) patients versus healthy non-CA subjects were common between the differential metabolites, plasma proteome, and lesional transcriptome. The plasma levels of 15 metabolites were different between CA patients and healthy non-CA analysis. Of these 15 metabolites, five were only integrated with KEGG pathways dysregulated in the lesional transcriptome of CA. The overlap analytic comparisons of enriched-KEGG pathways from these differential metabolites and the differential plasma proteome and lesional transcriptome identified 11 common pathways ($p<0.05$, false discovery rate corrected; Bayes factor>3).

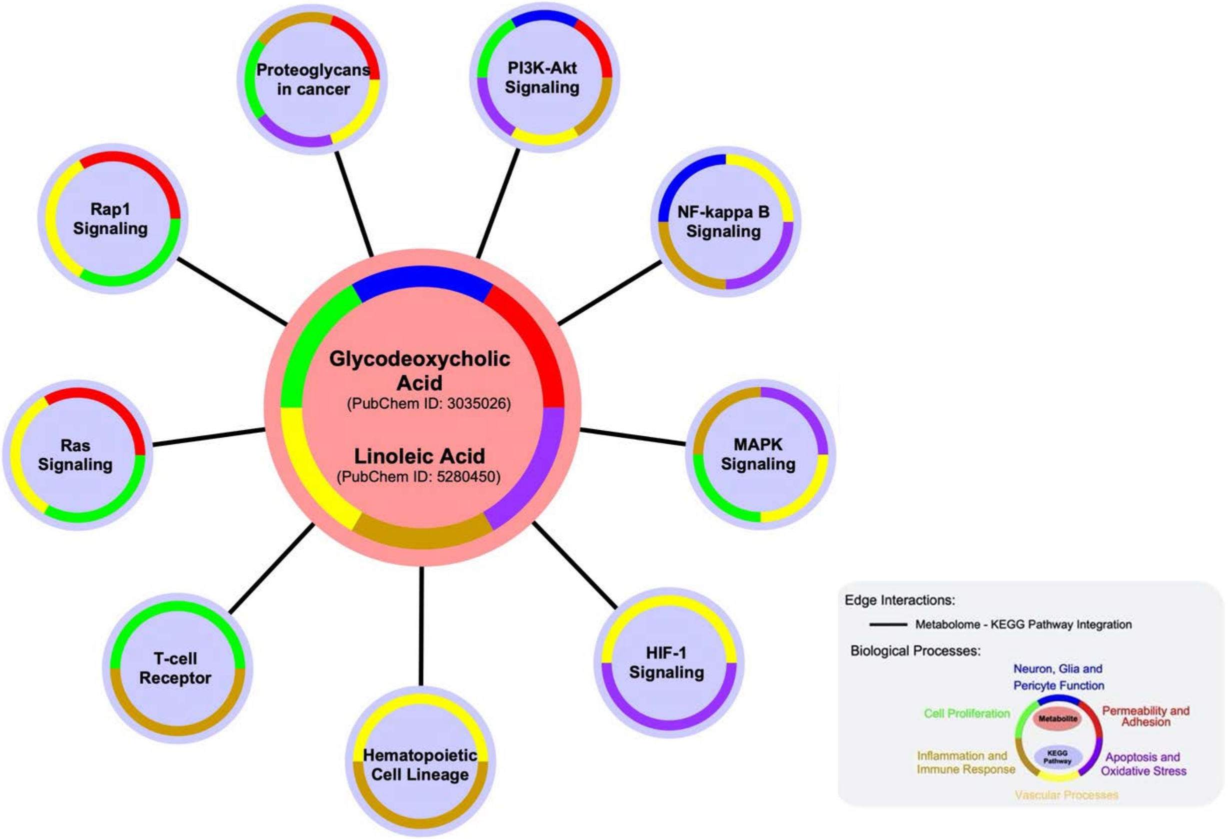

Supp. Fig. 4. Nine Kyoto Encyclopedia of Genes and Genomes (KEGG) pathways identified between cavernous angioma with symptomatic hemorrhage (CASH) and cavernous angioma without symptomatic hemorrhage (non-CASH) patients overlapped across differential metabolites, the differential plasma proteome, and lesional transcriptome of cavernous angioma disease. The plasma levels of glycodeoxycholic and linoleic acids were also different between CASH and non-CASH patients. Overlap analyses between these differential metabolites, the plasma proteome, and lesional transcriptome found nine KEGG pathways with CA relevance ($p<0.05$, false discovery rate corrected; Bayes factor>3).

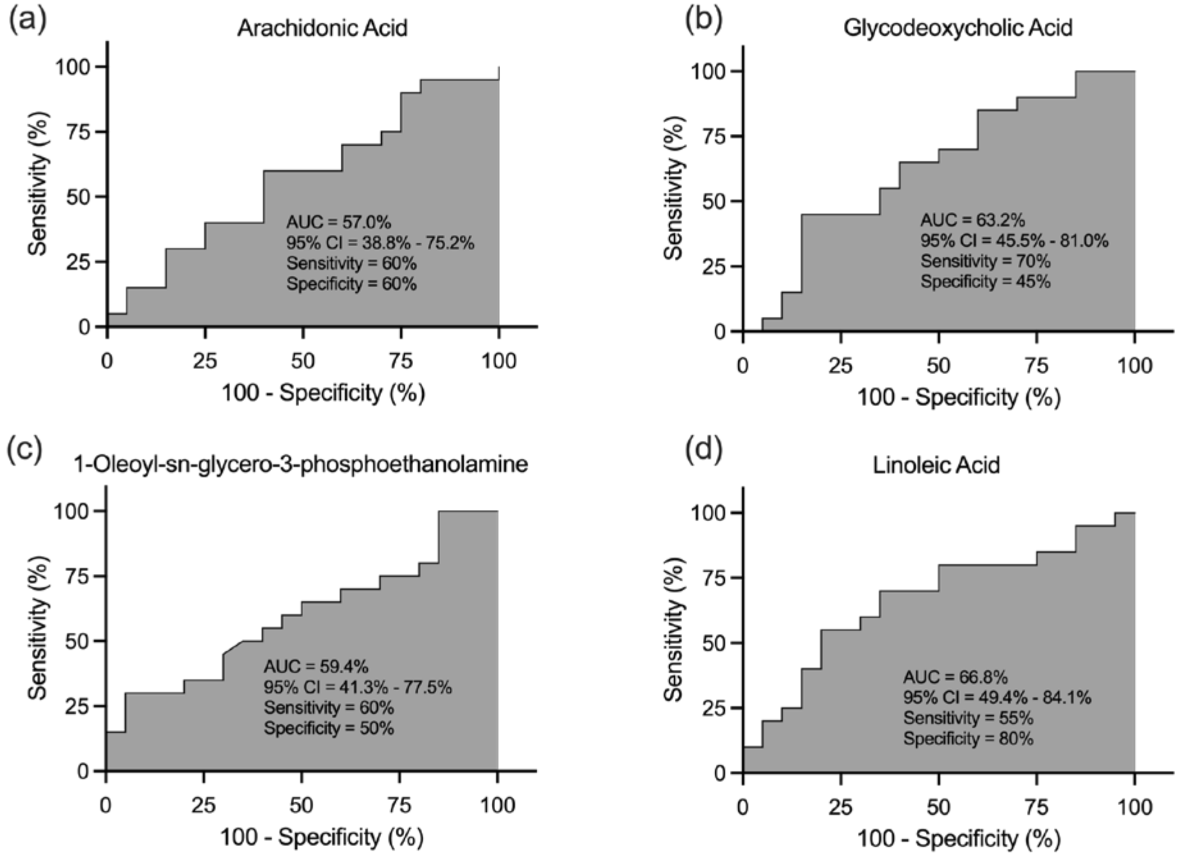

Supp. Fig. 5. Four metabolites differentially expressed in cavernous angioma with symptomatic hemorrhage (CASH) events were validated in an independent cohort. The four metabolites were validated in an independent cohort of (n=20 biologically independent CASH patients) vs. (n=20 biologically independent non-CASH patients). The receiving operating characteristic analyses showed that the ratio plasma levels of (**a**) arachidonic acid were able to differentiate CASH patients with 60% sensitivity and 60% specificity (area under the curve [AUC]=57.0%), (**b**) glycodeoxycholic acid distinguished CASH with 70% sensitivity and 45% specificity (AUC=63.2%), (**c**)1-oleyl-sn-glycero-3-phosphethanolamine performed with 60% sensitivity and 50% specificity (AUC=59.4%) while (**d**) linoleic acid showed 55% sensitivity and 80% specificity (AUC=66.8%).

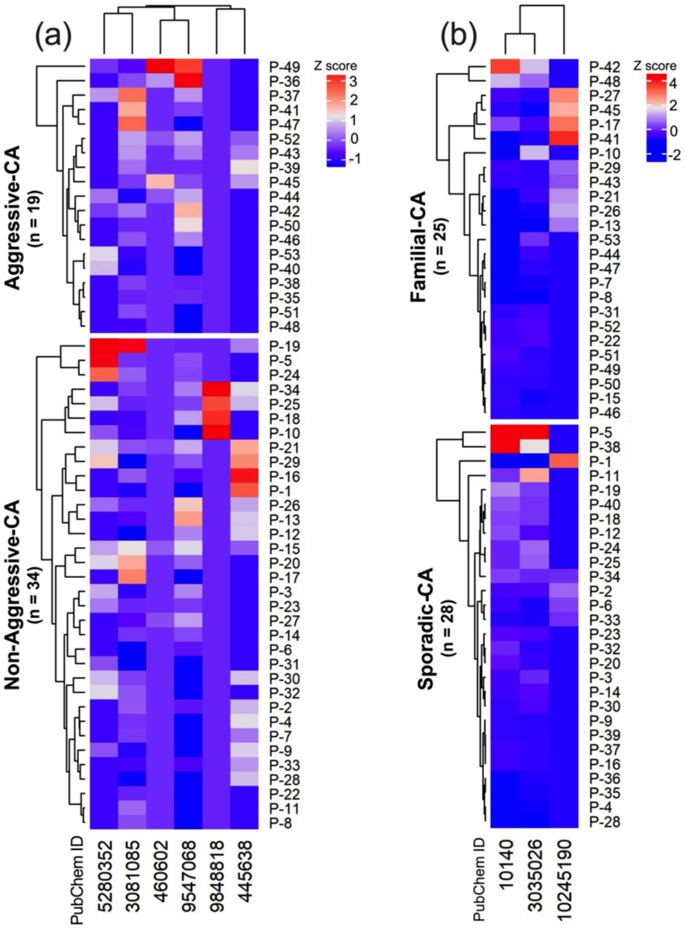

Supp. Fig. 6. Heatmaps of the differential metabolomic profiles in the clinical manifestations of cavernous angioma (CA) disease. A PLS-Discriminant Analysis showed differences in plasma levels of (**a**) six metabolites between aggressive (n=19 biologically independent patient samples) and non-aggressive (n=34 biologically independent patient samples) CA patients. (**b**) In addition, the plasma levels of three metabolites were different between familial (n=25 biologically independent patient samples) and sporadic (n=28 biologically independent patient samples) CA patients (all: $p<0.05$, FDR corrected; Bayes factor>3). Z-scores were calculated to compare metabolite levels across patients within each comparison group, with lower z scores (darker blue) representing lower relative plasma levels of metabolites. Metabolites with listed PubChem IDs are identified in **Supp. Tables 5** and **6**.

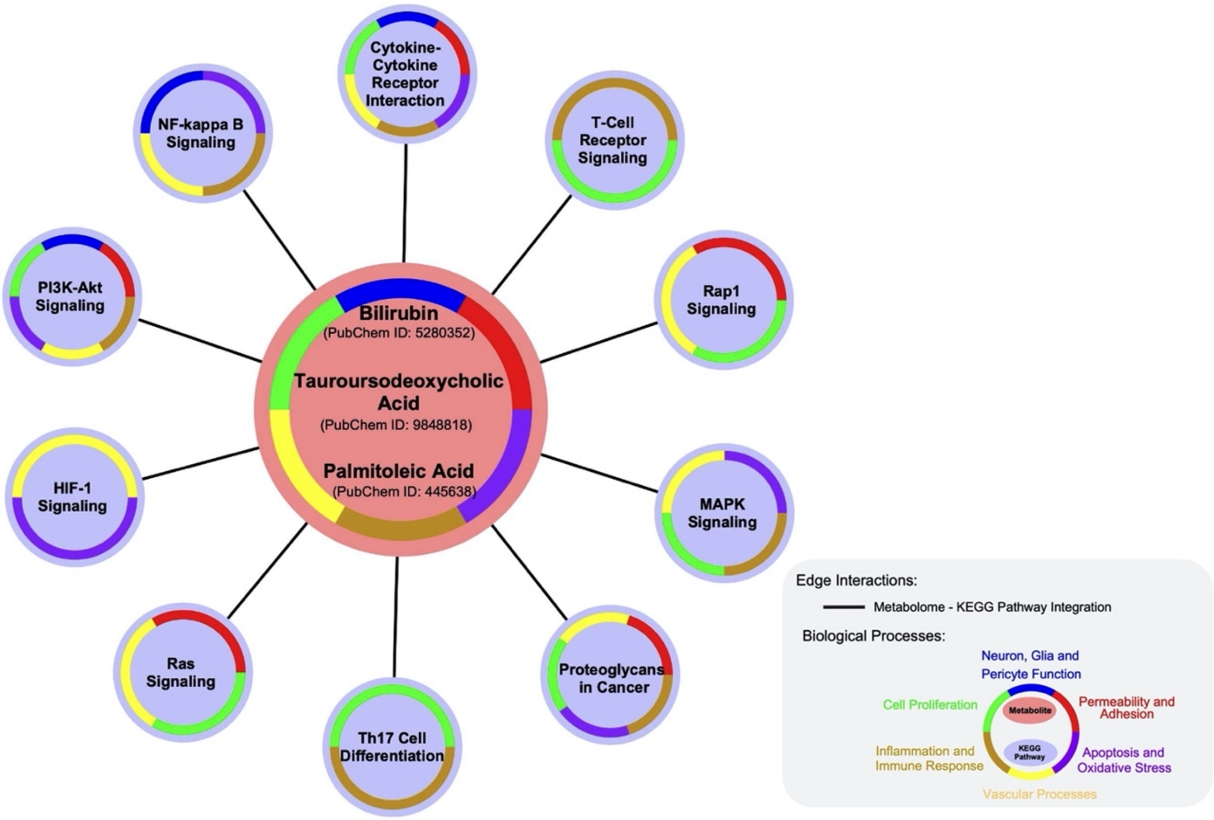

Supp. Fig. 7. Ten enriched- Kyoto Encyclopedia of Genes and Genomes (KEGG) pathways were identified to overlap between the differential metabolites identified between aggressive and non-aggressive cavernous angioma (CA) patients as well as the differential plasma proteome, and lesional transcriptome. The plasma levels of six metabolites were different between aggressive and non-aggressive CA patients. Ten KEGG pathways with CA relevance were found overlapping across three of the differential metabolites, the plasma proteome, and lesional transcriptome, that were then characterized into six CA biological processes (p<0.05, false discovery rate corrected; Bayes factor>3).

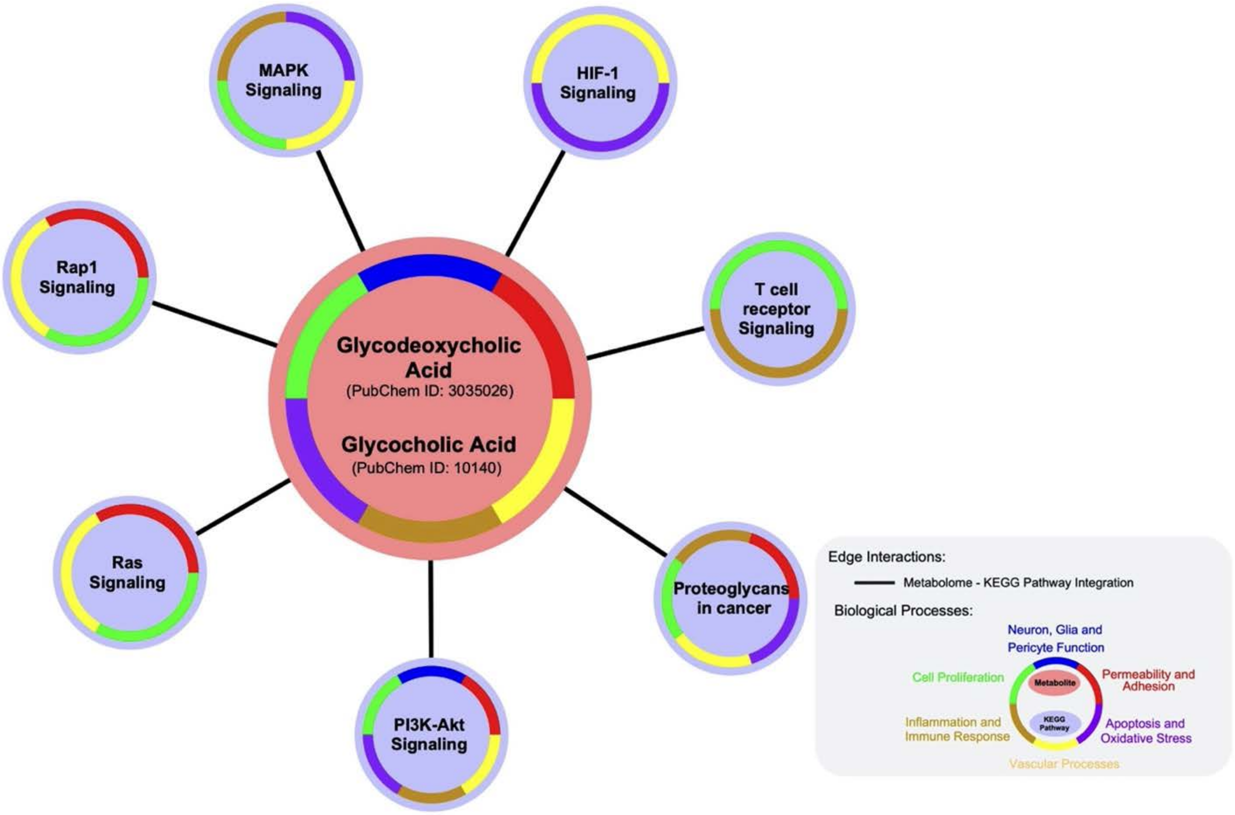

Supp. Fig. 8. Seven Kyoto Encyclopedia of Genes and Genomes (KEGG) pathways overlapped across differential metabolites, the differential plasma proteome, and lesional transcriptome when comparing familial- and sporadic-cavernous angioma (CA) patients. The plasma levels of glycodeoxycholic and glycocholic acids were different between familial-CA and sporadic-CA patients. Seven CA-relevant KEGG pathways overlapped across these two metabolites, the differential plasma proteome, and the lesional transcriptome ($p<0.05$, false discovery rate corrected; Bayes factor>3).

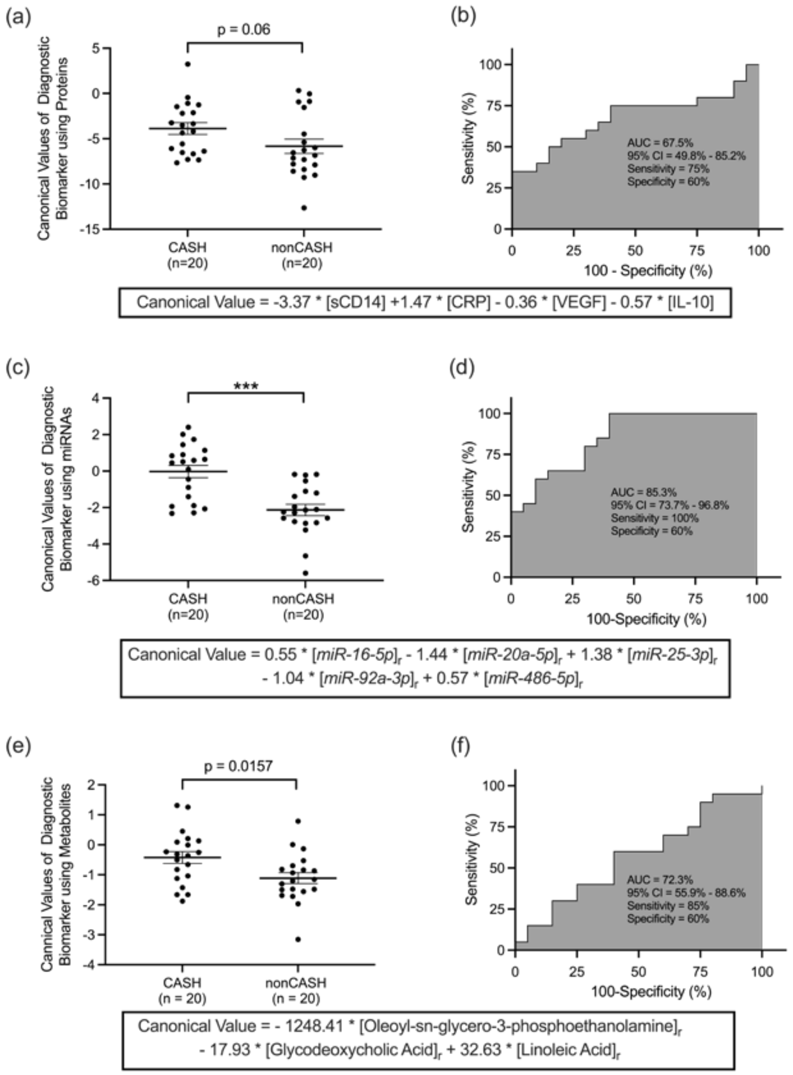

Supp. Fig. 9. Weighted combinations of plasma proteins, metabolites and micro-RNAs (miRNAs) separately to diagnose a cavernous angioma with symptomatic hemorrhage (CASH) event. The canonical values estimated by the best weighted combination with only plasma proteins were higher (**a-b**) in CASH (n=20 biologically independent patient samples) compared to cavernous angioma without symptomatic hemorrhage (non-CASH, n=20 biologically independent patient samples) patients (unpaired student T test, *p*=0.06) and distinguished these patients with 75% sensitivity and 60% specificity (area under the curve [AUC]=67.5%). Canonical values calculated for the optimal weighted combination of miRNA differentially expressed and validated in CASH were (**c**) higher in CASH compared to non-CASH patients (unpaired student T test, *p*<0.001), and (**d**) distinguished with 100% sensitivity and 60% specificity (AUC=85.3%). Finally, the optimal weighted combination of metabolites showed **(e)** higher canonical values in CASH patients (unpaired student T test, *p*=0.0157) and performed with (**f**) 85% sensitivity and 60% specificity (AUC=72.3%). CI, confidence interval; ***, *p*<0.001.
Error bars represent standard error of the mean.

Supplemental Table 1. Demographic characteristics and lesion features of human subjects in the discovery cohort.

Patient Characteristics	Patients with Cavernous Angioma (CA)		Healthy non-CA subjects
	CA with Symptomatic Hemorrhage (CASH)	CA without Symptomatic Hemorrhage (Non-CASH)	
Sample Size	5	48	17
Age (mean ± SD)	41.6 ± 20.3	47.6 ± 14.6	51.60 ± 20.38
Range years	27-77	19-80	20.70 – 81.19
Female %	80%	64.6%	82.35
Lesion Characteristics			
Number of susceptibility-weighted imaging lesions (mean ± SD)	1	14.15 ± 28.41	NA
Range	1	0 - 135	
Number of T_2-weighted lesions (mean ± SD)	1	2.27 ± 2.22	NA
Range	1	0 - 9	
Clinical Symptoms			NA
No prior cavernous angioma with symptomatic hemorrhage (CASH), n (%)	0%	56.25%	
CASH in prior year n (%)	100%	0%	
Prior CASH number (mean ± SD)	1	0.69 ± 0.97	
Age at symptom presentation (mean± SD)	39.0 ± 21.4	35.66 ± 15.79	
Phenotype, n (%)			NA
Sporadic	5 (100%)	23 (47.9%)	
CCM1	0 (0%)	9 (18.8%)	
CCM2	0 (0%)	5 (10.4%)	
CCM3	0 (0%)	2 (4.2%)	
Multifocal unknown genotype	0 (0%)	9 (18.8%)	
Ethnicity, n (%)			
White/European	4 (80%)	42 (87.5%)	6 (35.3%)
African American	1 (20%)	1 (2.1%)	9 (52.9%)
Hispanic of Mexican descent	0 (0%)	1 (2.1%)	0 (0%)
Asian	0 (0%)	0 (0%)	1 (5.9%)
Others	0 (0%)	4 (8.3%)	1 (5.9%)

Supplemental Table 2. The plasma levels of fifteen metabolites were different between cavernous angioma (CA) and healthy non-CA subjects ($p<0.05$, FDR corrected)

Plasma compound name	PubChem ID	CA patients (Normalized Mean ± SD $x10^{-4}$)	Healthy non-CA subjects (Normalized Mean ± SD $x10^{-4}$)	Sensitivity (%)	Specificity (%)	Accuracy (%)
1-(9Z-Octadecenoyl)-sn-glycero-3-phosphocholine	16081932	0.87±5.15	N/A	100	0	52.6
Linoleic Acid	5280450	25.44±22.14	14.44±17.00	60	78	68.4
1-Palmitoyl-2-hydroxy-sn-glycero-3-phosphoethanolamine	9547069	16.39±12.86	21.49±10.80	65	67	65.8
Cholic acid	221493	2.97±6.09	10.70±28.88	78	22	51.3
cis-9-Hexadecenoic acid	445638	3.72±4.95	2.21±3.67	45	89	65.8
Etiocholanedione	440114	1.40±3.09	2.68±4.50	75	33	55.3
Glycocholic acid	10140	14.96±24.08	3.97±4.46	55	67	60.5
Glycodeoxycholic acid	3035026	46.01±62.10	24.62±20.00	55	67	60.5
Deoxycholic acid	222528	1.15±3.10	6.06±19.33	100	0	52.6
Hydroxypalmitic acid	301590	8.72±8.36	5.88±6.16	63	67	64.5
Hypoxanthine	135398638	7.18±6.18	10.58±5.63	75	44	60.5
LysoPE 18:2	53480926	114.19±52.37	92.08±72.47	93	22	59.2
Phenylalanylphenylalanine	65104	0.77±4.13	N/A	100	0	52.6
Tauroursodeoxycholic acid	9848818	0.60±2.16	N/A	100	0	52.6
Urobilin	5280819	0.22±1.13	2.58±8.28	98	11	56.6

*N/A = Not Detectable

Supplemental Table 3. Four metabolites were differentially present in the plasma of (A) discovery cohort (p<0.05, FDR corrected), (B) independent propensity matched validation cohort (p<0.1, FDR corrected) of cavernous angioma with symptomatic hemorrhage (CASH) vs. cavernous angioma without symptomatic hemorrhage (non-CASH) patients

A

Plasma compound name	PubChem ID	CASH (Normalized Mean ± SD x10^{-4})	Non-CASH (Normalized Mean ± SD x10^{-4})
Linoleic Acid	5280450	26.16±22.46	16.67±15.09
1-Oleoyl-sn-glycero-3-phosphoethanolamine	9547071	N/A	2.27±3.19
Arachidonic acid (cis-5,8,11,14-Eicosatetraenoic acid)	444899	11.77±5.04	7.64±3.42
Glycodeoxycholic acid	3035026	27.20±21.14	47.54±64.06

B

Plasma compound name	PubChem ID	CASH (Normalized Mean ± SD x10^{-4})	Non-CASH (Normalized Mean ± SD x10^{-4})
Linoleic Acid	5280450	316.58±176.68	201.24±103.86
1-Oleoyl-sn-glycero-3-phosphoethanolamine	9547071	8.33±3.07	10.20±4.21
Arachidonic acid (cis-5,8,11,14-Eicosatetraenoic acid)	444899	92.57±38.69	78.37±27.50
Glycodeoxycholic acid	3035026	233.24±178.82	326.58±231.47

*N/A = Not Detectable

Supplemental Table 4. Demographic characteristics of the propensity matched validation cohort including 20 cavernous angioma with symptomatic hemorrhage (CASH) and 20 cavernous angioma without symptomatic hemorrhage (non-CASH) patients

	CASH	non-CASH	*p*-value
Sample Size	20	20	
Age at enrollment<br>Mean ± SD<br>Range	37.9 ± 10.6<br>[18.0 - 60.0]	36.7±10.6<br>[18.2 - 59.8]	*p*=0.72
Gender, n (%)			
Female	15 (75.0)	14 (70.0)	*p*>0.99
Male	5 (25.0)	6 (30.0)	
Phenotype, n (%)			
Sporadic	8 (40.0)	8 (40.0)	*p*>0.99
Familial	12 (60.0)	12 (60.0)	
Brainstem location, n (%)			
Yes	5 (25.0)	5 (25.0)	*p*>0.99
No	15 (75.0)	15 (75.0)	

Supplemental Table 5. Six metabolites had different plasma levels in aggressive compared to non-aggressive cavernous angioma patients ($p<0.05$, FDR corrected)

Plasma compound name	PubChem ID	Aggressive (Normalized Mean ± SD $x10^{-4}$)	Non-Aggressive (Normalized Mean ± SD $x10^{-4}$)
1-Palmitoyl-sn-glycero-3-phosphocholine	460602	10.90±31.66	0.57±1.88
1-Stearoyl-2-hydroxy-sn-glycero-3-phosphoethanolamine	9547068	10.76±9.67	6.55±6.43
7a-Hydroxy-3-oxo-4-cholestenoic acid (7alpha-Hydroxy-3-oxo-4-cholestenoic acid)	3081085	11.54±4.46	9.69±4.86
Bilirubin	5280352	5.09±8.31	11.97±15.90
cis-9-Hexadecenoic acid	445638	1.45±2.95	4.99±5.37
Tauroursodeoxycholic acid	9848818	N/A	0.94±2.64

*N/A = Not Detectable

Supplemental Table 6. Three metabolites were differentially present in the plasma of familial- compared to sporadic- cavernous angioma (CA) patients ($p<0.05$, FDR corrected)

Plasma compound name	PubChem ID	Familial-CA (Normalized Mean ± SD $x10^{-4}$)	Sporadic-CA (Normalized Mean ± SD $x10^{-4}$)
Decanoyl-L-carnitine	10245190	3.58±5.48	1.14±3.14
Glycocholic acid	10140	10.08±19.42	19.32±26.84
Glycodeoxycholic acid	3035026	33.09±33.87	57.54±77.43
