# Supplementary material for: Plasma metabolites with mechanistic and clinical links to the neurovascular disease cavernous angioma
Source: Commun Med (Lond). 2023 Mar 3;3:35. doi: 10.1038/s43856-023-00265-1 (PMC9984539; doi:10.1038/s43856-023-00265-1)
Supplement: Supplementary file 9 — Description of Additional Supplementary Files [file 43856_2023_265_MOESM9_ESM.pdf]

Description of Additional Supplementary Files

File Name: Supplementary Data 1

Description: Mass Spectrometry Characteristics for Differential Metabolites

File Name: Supplementary Data 2

Description: Nineteen plasma proteins were preselected based on previously defined relevance to cavernous angioma (CA) disease

File Name: Supplementary Data 3

Description: Source data for Figure 2, a and b, heatmaps of the differential metabolomic profiles in cavernous angioma (CA) disease and its clinical manifestations

File Name: Supplementary Data 4

Description: Source data for Figure 3 (integrative analyses of cholic acid and hypoxanthine between the differential microbiome, plasma proteome and lesional transcriptome of cavernous angioma (CA) disease) and Figure 4 (Arachidonic acid links *Enterobacter cloacae* and 11 Kyoto Encyclopedia of Genes and Genomes (KEGG) pathways enriched across the differential proteome and transcriptome of cavernous angioma with symptomatic hemorrhage (CASH) patients**)**

File Name: Supplementary Data 5

Description: The overlap of enriched-KEGG pathways between the transcriptome, proteome, and metabolites

File Name: Supplementary Data 6

Description: Nineteen plasma miRNAs were differentially expressed between CASH and cavernous angioma without symptomatic hemorrhage (non-CASH) patients included in the propensity matched scored

File Name: Supplementary Data 7

Description: Source data for Figures 5, Weighted combinations of plasma ratio of metabolites and micro-RNAs (miRNAs) improve the performance of the proteins-based cavernous angioma with symptomatic hemorrhage (CASH) biomarker
