# Supplementary material for: Plasma metabolites with mechanistic and clinical links to the neurovascular disease cavernous angioma
Source: Commun Med (Lond). 2023 Mar 3;3:35. doi: 10.1038/s43856-023-00265-1 (PMC9984539; doi:10.1038/s43856-023-00265-1)
Supplement: Supplementary file 10 — Reporting Summary [file 43856_2023_265_MOESM10_ESM.pdf]

nature portfolio

Corresponding author(s): Issam A. Awad

Last updated by author(s): Jan 20, 2023

Reporting Summary

Nature Portfolio wishes to improve the reproducibility of the work that we publish. This form provides structure for consistency and transparency in reporting. For further information on Nature Portfolio policies, see our Editorial Policies and the Editorial Policy Checklist.

Statistics

For all statistical analyses, confirm that the following items are present in the figure legend, table legend, main text, or Methods section.

n/a	Confirmed	
☐	☒	The exact sample size (*n*) for each experimental group/condition, given as a discrete number and unit of measurement
☐	☒	A statement on whether measurements were taken from distinct samples or whether the same sample was measured repeatedly
☐	☒	The statistical test(s) used AND whether they are one- or two-sided<br>*Only common tests should be described solely by name; describe more complex techniques in the Methods section.*
☐	☒	A description of all covariates tested
☐	☒	A description of any assumptions or corrections, such as tests of normality and adjustment for multiple comparisons
☐	☒	A full description of the statistical parameters including central tendency (e.g. means) or other basic estimates (e.g. regression coefficient) AND variation (e.g. standard deviation) or associated estimates of uncertainty (e.g. confidence intervals)
☐	☒	For null hypothesis testing, the test statistic (e.g. *F*, *t*, *r*) with confidence intervals, effect sizes, degrees of freedom and *P* value noted<br>*Give P values as exact values whenever suitable.*
☒	☐	For Bayesian analysis, information on the choice of priors and Markov chain Monte Carlo settings
☐	☒	For hierarchical and complex designs, identification of the appropriate level for tests and full reporting of outcomes
☐	☒	Estimates of effect sizes (e.g. Cohen's *d*, Pearson's *r*), indicating how they were calculated

Our web collection on statistics for biologists contains articles on many of the points above.

Software and code

Policy information about availability of computer code

Data collection

A one-to-one mapping with the single nearest neighbor was performed to match cavernous angioma with symptomatic hemorrhage (CASH) and non-CASH pairs using STATA version 16.0 (College Station, TX, USA).

Liquid chromatography-tandem mass spectrometry data acquisition for plasma metabolites in the discovery cohort was performed using an ultra-high performance Thermo Dionex Ultimate 3000 UHPLC (ThermoFisher, Waltham, MA, USA) coupled to an ultra-high resolution quadrapole time of flight Bruker Daltonics MaXis HD (Billerica, MA, USA) mass spectrometer. The data acquisition was performed using electrospray ionization in positive mode. Data collection was performed using a complete a lock mass correction using hexakis (1H, 1H, 2H-difluoroethoxy) phosphazene (Synquest Laboratories, Alachua, FL, USA) implemented within the Bruker Data Analysis Software. Feature detection was done with MZmine v2.38 software (http://mzmine.github.io/changelog.html). The MS2 file and quantification table were used in the Global Natural Products Social Molecular Networking feature based molecular networking workflow to get both networks and annotations for the metabolites. Discovery cohort metabolomic quantifications were performed at the Center for Microbiome Innovation at the University of California San Diego.

The data collection for the independent propensity-matched validation cohort was conducted using MassHunter Profinder Analysis version B.10 software (Agilent) and confirmed by comparison with authentic standards. The metabolomic quantifications were performed at Host-Microbe Metabolomics Facility at the University of Chicago.

Plasma levels measurements were assessed using Bio-Rad MESO QuickPlex SQ 120 (Meso Scale Diagnostics), by measuring light signal when the electrochemiluminescent labels are stimulated by electricity. The assessment was performed at the Flow Cytometry Core Facility at the University of Chicago.

Data analysis	The unsupervised differential metabolome was assessed with PLS-Discriminant Analysis (PLS-DA) using the R Software.<br><br>The interacting genes associated with the differential metabolites identified in the metabolome of cavernous angioma (CA) patients (p<0.05, FDR corrected) were queried using the Comparative Toxicogenomics Database (http://ctdbase.org/).<br><br>The enriched-KEGG pathways (p<0.05, FDR corrected; Bayes Factor>3) associated with the upregulated and downregulated DEGs (p<0.05, FDR corrected) within the human lesional transcriptome of NVUs (Gene Expression Omnibus #GSE123968) microdissected from CA lesions were queried independently using Lynx (http://lynx.ci.uchicago.edu).<br><br>For the proteome, the coding genes of the plasma proteins with a documented role in CA disease were first queried using Uniprot (https://www.uniprot.org/) and Genecards (https://www.genecards.org/). KEGG pathway enrichment analysis was then performed using Lynx. Finally, the common enriched-KEGG pathways between the differential plasma proteome and metabolome were identified using KEGG mapper (p<0.05, FDR corrected; Bayes>3).<br><br>The genomes of the bacterial gut species showing different relative abundances in CA disease were queried using KEGG mapper (p<0.05, FDR corrected; Bayes>3). The genes identified were further mapped into KEGG ortholog genes using AMON software to extract reactions involving the ortholog genes and metabolites of interest. Shotgun sequencing was also queried to further support evidence for the presence of a reaction link (AMON).

For manuscripts utilizing custom algorithms or software that are central to the research but not yet described in published literature, software must be made available to editors and reviewers. We strongly encourage code deposition in a community repository (e.g. GitHub). See the Nature Portfolio guidelines for submitting code & software for further information.

Data

Policy information about availability of data

All manuscripts must include a data availability statement. This statement should provide the following information, where applicable:

- Accession codes, unique identifiers, or web links for publicly available datasets
- A description of any restrictions on data availability
- For clinical datasets or third party data, please ensure that the statement adheres to our policy

All metabolomic data for the discovery cohort study are available in the GNPS repository, at https://gnps.ucsd.edu/ProteoSAFe/status.jsp?task=b82b664f25854df492aeec5420b95d45. All metabolomic data for the validation cohort study are available on the MassIVE repository, Accession number MSV000091098, https://massive.ucsd.edu/ProteoSAFe/dataset.jsp?task=d833d707661e432d91dc91c67d76d1ee. Data underlying all figures in the main manuscript are provided as source data files. Any additional records are available from the corresponding authors upon reasonable request.

Human research participants

Policy information about studies involving human research participants and Sex and Gender in Research.

Reporting on sex and gender	The term gender was used as a biological attribute (male/female) in the Methods and Supplemental Table 6. Findings in this study apply to combined sexes (male and female) or genders. Sex was considered for this study in order to have an equitable balance between groups. Sex (male/female) data are included in Supplemental Tables 1 and 6. Sex- and gender-based analyses were lacking because the sample size was not large enough for these analyses.
Population characteristics	Full population characteristics are provided in Supplemental Tables 1 and 6.
Recruitment	Patients were recruited during routine consultations or follow-up visits.
Ethics oversight	All subjects gave written informed consent in compliance with the Declaration of Helsinki, and the study was approved by the University of Chicago Institutional Review Board, which is guided by ethical principles consistent with the Belmont Report, and comply with the rules and regulations of the US Department of Health and Human Services Federal Policy for the Protection of Human Subjects (56 FR 28003).

Note that full information on the approval of the study protocol must also be provided in the manuscript.

Field-specific reporting

Please select the one below that is the best fit for your research. If you are not sure, read the appropriate sections before making your selection.

☒ Life sciences ☐ Behavioural & social sciences ☐ Ecological, evolutionary & environmental sciences

For a reference copy of the document with all sections, see nature.com/documents/nr-reporting-summary-flat.pdf

Life sciences study design

All studies must disclose on these points even when the disclosure is negative.

Sample size	The numbers of cases enrolled in discovery and validation cohorts are the largest ever reported in this rare disease, providing a first proof of concept for combined plasma metabolite, protein, and miRNA biomarkers for improved accuracy in specific clinical contexts of use. Because of these results, metabolomic discovery will now be incorporated in an ongoing large study sponsored by the U.S. National Institutes of Health (R01 NS114552) aimed at developing biomarkers of CASH, with a sample size powered to examine independent effects of multisite enrollment, age, sex, lesion location and genotype.
Data exclusions	Patients with partial or complete resection of CA or any prior brain irradiation were excluded. Healthy non-CA subjects were excluded if they had (a) any medical or neurologic condition requiring ongoing follow-up or medical treatment in the preceding year, (b) a history of concussion or brain trauma in the preceding year, (c) a history of prior brain irradiation at any time, (d) been pregnant or lactating in the preceding year, (e) used recreational, psychoactive, or neuroleptic drugs in the prior year.
Replication	The fragmentation pattern of the differential plasma metabolites identified between patients with a CASH event in the prior year and non-CASH patients was validated in an independent propensity matched validation cohort using a supervised LC-MS/MS approach.
Randomization	Allocation was not random. One hundred and nine consecutive CA subjects, enrolled between August 2016 and October 2020 included 20 CASH cases. These were best matched with 20 of the non-CASH cases for (1) age at enrollment, (2) gender (male/female), (3) phenotype (sporadic/familial), and (4) harboring brainstem lesion (yes/no).
Blinding	Blinding was not possible during group allocation, because the groups were already either CA with symptomatic hemorrhage, CA without symptomatic hemorrhage or healthy controls. Impossible to blind the allocation of groups whose subjects either already had or did not have CA disease.

Reporting for specific materials, systems and methods

We require information from authors about some types of materials, experimental systems and methods used in many studies. Here, indicate whether each material, system or method listed is relevant to your study. If you are not sure if a list item applies to your research, read the appropriate section before selecting a response.

Materials & experimental systems

n/a	Involved in the study	
☒	☐	Antibodies
☒	☐	Eukaryotic cell lines
☒	☐	Palaeontology and archaeology
☒	☐	Animals and other organisms
☐	☒	Clinical data
☒	☐	Dual use research of concern

Methods

n/a	Involved in the study	
☒	☐	ChIP-seq
☒	☐	Flow cytometry
☒	☐	MRI-based neuroimaging

Clinical data

Policy information about clinical studies
All manuscripts should comply with the ICMJE guidelines for publication of clinical research and a completed CONSORT checklist must be included with all submissions.

Clinical trial registration	NA
Study protocol	NA
Data collection	For this prospective study, a discovery cohort of 53 consecutive CA patients (25 familial-CA, and 28 sporadic-CA) was enrolled at a single referral center (www.uchicagomedicine.org/ccm) between April 2017 and August 2018, during routine consultations or follow-up visits. One hundred and nine consecutive CA subjects, enrolled between August 2016 and October 2020 included 20 CASH cases.
Outcomes	NA
